# A polyvalent DNA prime with matched polyvalent protein/GLA-SE boost regimen elicited the most robust and broad IgG and IgG3 V1V2 binding antibody and CD4+ T cell responses among 13 HIV vaccine trials

**DOI:** 10.1080/22221751.2025.2485317

**Published:** 2025-04-07

**Authors:** Zoe Moodie, Shuying Sue Li, Elena E. Giorgi, LaTonya D. Williams, One Dintwe, Lindsay N. Carpp, Shiyu Chen, Kelly E. Seaton, Sheetal S. Sawant, Lu Zhang, Jack Heptinstall, Shuying Liu, Nicole Grunenberg, Frank Tomaka, Supachai Rerks-Ngarm, Punnee Pitisuttithum, Sorachai Nitayaphan, Julie A. Ake, Sandhya Vasan, Giuseppe Pantaleo, Ian Frank, Lindsey R. Baden, Paul A. Goepfert, Michael Keefer, Mike Chirenje, Mina C. Hosseinipour, Kathryn Mngadi, Fatima Laher, Nigel Garrett, Linda-Gail Bekker, Stephen De Rosa, Erica Andersen-Nissen, James G. Kublin, Shan Lu, Peter B. Gilbert, Glenda E. Gray, Lawrence Corey, M. Juliana McElrath, Georgia D. Tomaras

**Affiliations:** a Vaccine and Infectious Disease Division, Fred Hutchinson Cancer Center, Seattle, WA, USA; b Center for Human Systems Immunology, Duke University School of Medicine, Durham, NC, USA; c Department of Surgery, Duke University School of Medicine, Durham, NC, USA; d Duke Human Vaccine Institute, Duke University School of Medicine, Durham, NC, USA; e Cape Town HVTN Immunology Laboratory, Hutchinson Centre Research Institute of South Africa, Cape Town, South Africa; f Worcester HIV Vaccine, Worcester, MA, USA; g Department of Research & Development, Janssen Vaccines and Prevention/Johnson & Johnson, Titusville, NJ, USA; h Thai Ministry of Public Health, Nonthaburi, Thailand; i Vaccine Trials Center, Faculty of Tropical Medicine, Mahidol University, Bangkok, Thailand; j Royal Thai Army, Armed Forces Research Institute of Medical Sciences, Bangkok, Thailand; k U.S. Military HIV Research Program, CIDR, Walter Reed Army Institute of Research, Silver Spring, MD, USA; l Henry M. Jackson Foundation for the Advancement of Military Medicine, Bethesda, MD, USA; m Service of Immunology and Allergy, Lausanne University Hospital and University of Lausanne, Lausanne, Switzerland; n Infectious Diseases Division, Perelman School of Medicine, University of Pennsylvania, PA, USA; o Department of Medicine, Brigham and Women's Hospital, Boston, MA, USA; p Department of Medicine, University of Alabama at Birmingham, Birmingham, AL, USA; q Department of Medicine, University of Rochester, Rochester, NY, USA; r College of Health Sciences Clinical Trials Research Centre, University of Zimbabwe, Harare, Zimbabwe; s UNC-Project Malawi, Lilongwe, Malawi; t Department of Medicine, UNC School of Medicine, University of North Carolina at Chapel Hill, Chapel Hill, NC, USA; u Clinical Research Division, Aurum Institute, Johannesburg, South Africa; v Perinatal HIV Research Unit (PHRU), Wits Health Consortium, Faculty of Health Sciences, University of the Witwatersrand, Soweto, Johannesburg, South Africa; w Desmond Tutu HIV Centre, University of Cape Town, Cape Town, South Africa; x Centre for the AIDS Programme of Research in South Africa (CAPRISA), University of KwaZulu-Natal, Durban, South Africa; y Discipline of Public Health Medicine, School of Nursing and Public Health, University of KwaZulu-Natal, Durban, South Africa; z Department of Medicine, University of Massachusetts Chan Medical School, Worcester, MA, USA; aa Public Health Sciences Division, Fred Hutchinson Cancer Center, Seattle, WA, USA; bb Department of Biostatistics, University of Washington, Seattle, WA, USA; cc South African Medical Research Council, Cape Town, South Africa; dd Department of Laboratory Medicine and Pathology, University of Washington, Seattle, WA, USA

**Keywords:** Binding antibody multiplex assay, cross-protocol analysis, Env V1V2 binding antibody response breadth score, intracellular cytokine staining, matched polyvalent DNA-polyvalent protein/GLA-SE prime-boost regimen

## Abstract

Developing an effective HIV vaccine is a momentous challenge. An exceptionally wide range of candidate HIV vaccines have been tested, yet many were poorly immunogenic, and of the select few that advanced into efficacy trials, only one demonstrated any efficacy. Here we report the results of the largest-scale cross-protocol immunogenicity comparison to date: 13 HIV vaccine trials (including 36 vaccine regimens) conducted across nine countries worldwide, strengthened by standardized trial designs, validated assays in centralized laboratories, and harmonized immunogenicity endpoints – providing an objective approach to identify the HIV vaccine candidate(s) with the best immunogenicity. A polyvalent DNA prime + protein boost regimen (HVTN 124) including Env immunogens of four subtypes, matched between prime and boost, achieved the best anti-V1V2 antibody responses by a large margin and also induced high CD4+ T-cell responses – two key immune responses implicated in HIV vaccine protection. Our results provide strong support to test this promising HIV vaccine design in more advanced phase clinical trials and will also guide the future design of additional HIV vaccines.

**Trial registration:** ClinicalTrials.gov identifier: NCT01799954..

**Trial registration:** ClinicalTrials.gov identifier: NCT02109354..

**Trial registration:** ClinicalTrials.gov identifier: NCT02404311..

**Trial registration:** ClinicalTrials.gov identifier: NCT02207920..

**Trial registration:** ClinicalTrials.gov identifier: NCT02296541..

**Trial registration:** ClinicalTrials.gov identifier: NCT03284710..

**Trial registration:** ClinicalTrials.gov identifier: NCT02915016..

**Trial registration:** ClinicalTrials.gov identifier: NCT02997969..

**Trial registration:** ClinicalTrials.gov identifier: NCT03122223..

**Trial registration:** ClinicalTrials.gov identifier: NCT03409276..

**Trial registration:** ClinicalTrials.gov identifier: NCT02968849..

**Trial registration:** ClinicalTrials.gov identifier: NCT03060629..

**Trial registration:** ClinicalTrials.gov identifier: NCT00223080..

## Introduction

Reported over a decade ago, the recombinant canarypox vector vaccine (ALVAC) prime and recombinant glycoprotein 120 subunit (alum-adjuvanted AIDSVAX B/E) boost HIV vaccine tested in the RV144 trial remains the only HIV vaccine regimen to have demonstrated any efficacy against HIV-1 acquisition: 31.2% [95% confidence interval (CI) 1.1 to 52.1; *P* = 0.04] against a diagnosis of HIV-1 acquisition over 3 years follow-up post last-dose at 6 months [1]. The prespecified immune correlates analysis identified IgG antibodies against variable regions 1 and 2 (V1V2) of the HIV-1 envelope protein (Env) as inverse correlates of risk (CoR) and IgA antibodies against Env as direct CoRs [2]. Follow-up studies also identified IgG antibodies to V2 and V3 linear epitopes [3], IgG antibodies to individual V1V2 antigens as well as cross-reactivity scores quantifying magnitude and breadth for V1V2 across multiple subtypes [4], IgG3 antibody response rates and magnitude to V1V2 [5], and polyfunctional Env-specific CD4^+^ T cells [6] as inverse CoRs, as well as suggested a potential role for Fc-mediated antibody function [7,8].

The RV144 results sparked efforts to elicit higher levels of immune responses implicated in protection, as well as more durable, qualitatively improved responses (e.g. greater breadth) with the hope of improving upon the modest vaccine efficacy of RV144 [9]. Multiple subsequent efficacy trials of various viral vector prime-protein boost regimens were conducted, but no efficacy was observed [10–13] – possibly owing to the low V1V2 antibody responses among most vaccine recipients in these efficacy trials, when evaluated [12,14,15]. Multiple trials were conducted to study platforms, protein inserts, protein doses, regimens, schedules, and adjuvants. Bivalent and polyvalent HIV vaccines were also developed [16,17] to address HIV-1 antigenic diversity. The RV144 findings also drove the development of a subtype C-adapted version of the RV144 regimen, thus customized for southern Africa, where subtype C predominates [18]. This regimen was tested in the HVTN 702 phase 2b/3 efficacy trial [10]. While that trial was halted due to meeting prespecified efficacy futility criteria, the correlates of risk analysis indicated that strong CD4+ T-cell responses, in conjunction with strong V1V2-specific IgG responses, were associated with a decreased risk of HIV-1 acquisition [15].

These trials, including the non-efficacious HVTN 702 and 705 trials, provide valuable data that allow comparisons of the regimens’ immunogenicity to identify the vaccine designs that may be most effective in eliciting immune correlates of protection. To maximize the scientific insights that could be drawn from these data, a cross-protocol immunogenicity comparison across 36 HIV-1 vaccine regimens tested in 13 HIV-1 vaccine trials, including three efficacy trials [RV144, HVTN 702, and Imbokodo (HVTN 705/HPX2008)] and a phase 1b trial (HVTN 097) evaluating the RV144 regimen in South Africa. In the context of the RV144 and HVTN 702 correlates results discussed above, the present analysis focused primarily on cross-trial comparisons of Env V1V2 binding antibody (bAb) response breadth, as well as CD4+ T-cell responses.

## Materials and methods

### Study design and participants

Details of each trial have been described previously: HVTN 096 [19], HVTN 097 [20], HVTN 100 [21], HVTN 105 [22], HVTN 106 [23], HVTN 107 [24], HVTN 108 [25], HVTN 111 [26], HVTN 120 [27], HVTN 124 [28], HVTN 702 [10], HVTN 705 [11], and RV144 [1]. All trials enrolled healthy adults aged 18–50 years not living with HIV at enrollment. Female participants agreed to consistent contraception; those pregnant and breastfeeding were excluded. Full eligibility criteria are described in the primary publications.

Randomization sequences were obtained by computer-generated random numbers and provided to each research site through the HVTN statistical and data management centre's web-based randomization system or for RV144 [1] through centrally generated randomization lists. The pharmacist with primary responsibility for dispensing study products maintained security of the treatment assignments.

### Ethics

All study participants provided written informed consent before participation. The trials were each approved by the research ethics committees of participating sites and all applicable country-specific regulators. All trials were registered with ClinicalTrials.gov and if applicable, the South African National Clinical Trials Registry and the Pan African Clinical Trials Registry. Specific details for each trial follow: Trial: Identifier, Date of Trial Registration (defined as “First Submitted” for ClinicalTrials.gov, unless otherwise stated). Ethics Committee(s) Granting Approval, Institution(s) of the Ethics Committee(s), and Reference, File, or Approval Number(s).

HVTN 096: NCT01799954, 2013-02-22. The study protocol was approved by the institutional ethics committee of Centre Hospitalier Universitaire Vaudois and by Swissmedic, the Swiss Agency for Therapeutic Products (Bern, Switzerland).

HVTN 097: NCT02109354, 2013-12-12. HVTN 097 was approved by the University of the Wits Human Research Ethics Committee (AUR1-3-122), the University of Witwatersrand, Johannesburg (121107), and the University of Cape Town Faculty of Health Sciences Human Research Ethics Committee (139/2013).

HVTN 100: NCT02404311, trial registration initiated prior to 2015-02-09 and achieved on 2015-03-31. South African National Clinical Trials Registry: DOH–27-0215-4796, trial registration initiated prior to 2015-02-09 and achieved on 2015-02-09. HVTN 100 was approved by the SAMRC Human Research Ethics Committee (EC013-9/2014), the Wits Human Research Ethics Committee (140705B), the University of Cape Town Faculty of Health Sciences Human Research Ethics Committee (443/2014), the University of Kwazulu-Natal Research Office Biomedical Research Ethics Administration (BFC301/14), and the University of Witwatersrand, Johannesburg Human Research Ethics Committee (Medical) (140705B).

HVTN 105: NCT02207920, 2014-07-31. HVTN 105 was approved by the Columbia Human Research Protection Office Institutional Review Boards (IRB-AAAN6603, IRB-AAAN6603), the Fred Hutch Institutional Review Board (8267), the Vanderbilt University Institutional Review Board (140828), the UCSF Human Research Protection Program Institutional Review Board (14-13672), the University of Pennsylvania Office of Regulatory Affairs Institutional Review Board (820330), and the University of Rochester Research Subjects Review Board (RSRB00052283).

HVTN 106: NCT02296541, 2014-11-18. The protocol was approved by the institutional review boards and biosafety committees at all sites (Brigham and Women’s Hospital; Fred Hutchinson Cancer Center; San Francisco Department of Public Health; The Fenway Institute; University of Alabama at Birmingham; Emory University; and Centre Hospitalier Universitaire Vaudois).

HVTN 107: NCT03284710, 2017-09-13. South African National Clinical Trials Registry: DOH-27-0715-4894, 2014-10-07. Initial and ongoing approvals of the study protocol and research review were provided by the Medicine Control Council (20141022), the Fred Hutchinson Cancer Research Center institutional review board (IRB), and local research ethics committees for each site: National Health Bioethics Committee (CNBS) (Ref 125/CNBS/2014), University of Cape Town Human Research Ethics Committee (Ref 790/2014), University of Witwatersrand Human Research Ethics Committee (Medical) (#141108), University of Kwazulu-Natal Biomedical Research Ethics Committee (Ref BFC453/14), University of Zimbabwe Joint Research Ethics Committee (Ref 186/15).

HVTN 108: NCT02915016, 2016-09-22. South African National Clinical Trials Registry: DOH-27-1015-5117. The trial was approved by research ethics committees of participating sites.

HVTN 111: NCT02997969, 2016-12-16. South African National Clinical Trials Registry: DOH-27–0715–4917. HVTN 111 was approved by the University of North Carolina at Chapel Hill Office of Research Ethics Biomedical IRB (14-3138), the Mbeya Medical Research and Ethics Committee (SZEC-2439/R.E/V.1/03), the University of Witwatersrand, Johannesburg Human Research Ethics Committee (Medical) (150212), the SAMRC Human Research Ethics Committee (EC011-3/2015), and the University of Witwatersrand, Johannesburg Human Research Ethics Committee (Medical) (150212).

HVTN 120: NCT03122223, 2017-02-14. The study was approved by the institutional review boards of Atlanta-Hope Clinic/Emory University, Boston-Brigham/Partners, Boston-Fenway, Case Western University, Vanderbilt University, University of Pennsylvania, University of Rochester, University of California San Francisco, and Fred Hutch Cancer Center in the United States; and Medical Research Council of Zimbabwe, University of Zambia Biomedical Research Ethics Committee, and Mbeya Medical Research and Ethics Committee in Africa.

HVTN 124: NCT03409276, 2018-01-09. Institutional biosafety committees and institutional review boards and ethics committees at each site approved the protocol.

HVTN 702: NCT02968849, 2016-11-01. HVTN 702 was approved by the University of Cape Town Human Faculty of Health Sciences Human Research Ethics Committee (622/2016, 623/2016); the South African Medical Research Council Ethics Committee (EC020-7/2016); the University of KwaZulu-Natal Biomedical Research Ethics Committee (BFC479/16); the University of the Witwatersrand, Johannesburg Human Research Ethics Committee (Medical) (160208B); and the Sefako Makgatho University Research Ethics Committee (SMUREC/P/192/2016:CR).

HVTN 705: NCT03060629, 2017-02-17. The protocol, protocol amendments, and other relevant documents were approved by the following institutional review boards, ethics committees, and applicable regulatory entities: Malawi – Malawi National Health Sciences Research Committee, University of North Carolina IRB (due to site academic affiliation); Mozambique – Mozambique INS Health Bioethics Institutional Committee; South Africa: University of Witwatersrand Human Research Ethics Committee, University of Cape Town Human Research Ethics Committee, South Africa Medical Research Council Ethics Committee, University of KwaZulu-Natal Biomedical Research Ethics Committee, University of Pretoria Research Ethics Committee, Sefako Makgatho University Research Committee; Zambia – University of Zambia Biomedical Research Ethics Committee, University of Alabama IRB (due to site academic affiliation), Emory University IRB (due to site academic affiliation); and Zimbabwe – University of Zimbabwe Joint Research Ethics Committee.

RV144: NCT00223080, 2005-09-13. The protocol was reviewed by the Ethical Review Committee for Research in Human Subjects, Ministry of Public Health, Thailand [Federalwide Assurance Number (FWA): FWA00001953; Reference IRB number: 91/2545]; the Institutional Review Board of the Royal Thai Army Medical Department (FWA00001813, 5005 h/45); the Institutional Review Board of Mahidol University (FWA 00000926, MU-CIRB 2008/029.0404); and the Office of Human and Animal Research Oversight, U.S. Army Medical Research and Development Command (E00378.43a/RV144/WRAIR#900). It was also independently reviewed and endorsed by the World Health Organization and the Joint United Nations Program on HIV/AIDS and by the AIDS Vaccine Research Working Group of the National Institute of Allergy and Infectious Diseases at the National Institutes of Health.

### Immunogenicity assays

Immune response data are available on 1688 per-protocol participants who were not diagnosed with HIV-1 and received all scheduled HIV vaccinations prior to the primary immunogenicity blood draw. All assays were performed blinded to vaccine group. Measurements included IgG and IgG3 binding antibodies (bAbs) and T-cell responses.

#### Binding antibody multiplex assay (BAMA)

Assayed serum samples were collected pre-vaccination and at 2 weeks (4 weeks for HVTN 705) after the final primary series HIV immunization. Serum HIV-1-specific IgG and IgG3 bAb responses were measured against V1V2 antigens (listed in Tables S1 and S2) on a Bio-Plex instrument (Bio-Rad) using a validated custom HIV-1 Luminex assay [29]. To assess total HIV-1 specific responses, serum IgG and IgG3 bAb responses were also measured against Con 6 gp120/B (a consensus clade B gp120 protein). All BAMA assays were performed in the Tomaras lab (Duke University).

The readout was obtained as the background-subtracted mean fluorescence intensity (MFI), where the background included both an antigen-specific plate level control (i.e. a blank well containing antigen-coated beads run on each plate) and a specimen-specific control (i.e. a serum well containing blank beads).

Samples were considered positive if they met the following criteria: (1) net MFI ≥ antigen-specific positive response threshold (defined separately for each trial as the maximum of 100 and the 95th percentile of pre-vaccination net MFI values), (2) net MFI > 3 times the baseline net MFI, and (3) MFI > 3 times the baseline MFI.

IgG responses were assayed at a dilution of 1:50 and IgG3 responses were assayed at a dilution of 1:40 (except for HVTN 096 at a dilution of 1:50) against gp70-V1V2 antigens.

Further details are in the Supplementary Methods.

#### Intracellular cytokine staining assay (ICS)

Peripheral blood mononuclear cells collected at 2 weeks (4 weeks for HVTN 705) after the final primary series HIV immunization were isolated and cryopreserved from whole blood [30]. HIV-1-specific CD4 + and CD8+ T-cell responses to vaccine-matched peptide pools or potential T cell epitope (PTE) global peptide pools (Table S4) were measured by intracellular cytokine staining using a validated flow cytometry assay [31,32]. ICS assays were run by the HVTN Laboratory Center in either the Seattle (Fred Hutchinson Cancer Center) or the Cape Town (Cape Town HVTN Immunology Laboratory) laboratory; assay concordance has been demonstrated between the two laboratories [33].

Cryopreserved PBMCs were stimulated with synthetic HIV-1 Envelope peptide pools. DMSO, the diluent for the peptide pools, served as the negative control (run in duplicate), while cells stimulated with staphylococcal enterotoxin B (SEB), a polyclonal stimulant, served as the positive control. The background-adjusted percentage of CD4 + and CD8+ T cells expressing IFN-γ and/or IL-2 was analyzed, where this net percent was calculated as % of antigen-stimulated cells minus % of unstimulated, negative control cells.

A positive response was determined based on a one-sided Fisher's exact test, comparing the frequency of IFN-γ and/or IL-2-producing cells in the peptide-stimulated well to that in the negative control wells. Details are in the Supplementary Methods.

If any Env sub-peptide pools showed a positive response for a T-cell subset, the overall “Any Env” response for that T-cell subset was considered positive and the Any Env magnitude was defined as the maximum response magnitude observed across all Env peptide pools tested in the trial. The same approach was applied in determining responses for Any Gag. Details are in the Supplementary Methods.

### Statistical methods

Immunogenicity analyses included per-protocol participants. Participants with an HIV-1 positive test by the primary immunogenicity time point or samples that failed assay-specific quality-control criteria were excluded. For HVTN 097, data were combined across the two treatment arms: T consists of T1 and T2, where T1 was given tetanus and T2 was given placebo at day 0.

As bAb responses were measured across different sets of antigens for each trial, we defined a participant-level summary measure for cross-trial comparisons. The participant-level bAb breadth score for a given isotype was defined as the geometric mean of responses to the 3 heterologous V1V2 antigens with the highest median responses among all participants by regimen. If fewer than 3 such antigens were measured, the breadth score was based on all available heterologous V1V2 antigens.

T-cell responses were also measured across different sets of peptide pools therefore comparisons were made based on summary measures. CD4 + and CD8+ T-cell magnitudes to Any Env were defined as the maximum over all Env peptide pools tested.

Positive response rates between two different regimens were compared using Barnard’s exact test. Response levels, including breadth scores, of all vaccine recipients were compared using Wilcoxon rank sum tests with 95% CIs calculated using the score test method [34]. *P*-values < 0.05 were judged statistically significant. No adjustment for multiple comparisons was done. Due to low response levels, CD8+ T-cell responses were not formally compared.

Analyses were performed using R statistical software (version 4.0.4; R Foundation for Statistical Computing, Vienna, Austria).

## Results

### Trials and vaccine regimens included in the analysis

Each of the 13 Phase 1–3 trials of 36 HIV-1 vaccine regimens (Table 1) was a double-blind, randomized clinical trial of healthy adults without HIV-1 at enrollment. Twelve were conducted by the HIV Vaccine Trials Network (HVTN), whereas the RV144 trial was conducted by the U.S. Military HIV Research Program. The trials were conducted between 2003 and 2018 at study sites in Malawi, Mozambique, South Africa, Switzerland, Tanzania, Thailand, the United States, Zambia, and Zimbabwe (Figure S1). Apart from HVTN 107, each trial was placebo-controlled. The regimens differed by delivery system, immunogen sequence, protein dose, adjuvant, and schedule (Table 1). Immune response data were available on 1688 participants. Within each trial, the proportion of female participants with immunogenicity data ranged from 40% to 67%, except in HVTN 705 and HVTN 702, where immune responses were only measured in a subset of female participants by design (Table S5). Across the trials that collected data on race and ethnicity (all except RV144), the majority of participants with immunogenicity data were Black/African American (1053/1487; 70.8%) and 344/1487 (23.1%) were White (Table S5). Numbers for other races are reported in Table S5. Approximately 80% or more of participants in each trial with immunogenicity data were aged between 18 and 35 years, except HVTN 106 which enrolled 60.3% aged between 18 and 35 years.

**Table 1.** Description of trial regimens by country, study start date, platforms, proteins, adjuvants, doses, and number and timing of immunizations.

Trial (phase)	Country/ies^1^	Start	Platform(s)	Platform inserts^2^ (Subtype: insert)	Protein	Protein inserts (Subtype: insert)	Total prot dose	Adj.	Arm (n^3^)	Schedule (Months)^4^
RV144 (3)	THA	09/2003	Canarypox	E: 92TH023gp120; B: gp41^5^, gag, pro	AIDSVAX B/E gp120	B: MN; E: A244	600 µg	Alum	T (8202)	ALVAC only: 0, 1; ALVAC + prot/alum: 3, 6
097 (1)	RSA	06/2013	Canarypox	E: 92TH023gp120; B: gp41^5^, gag, pro	AIDSVAX B/E gp120	B: MN; E: A244	600 µg	Alum	T (80)^6^	ALVAC only: 1, 2; ALVAC + prot/alum: 4, 7
100 (1-2)	RSA	02/2015	Canarypox	C: ZM96 gp120; B: gp41^5^, gag, pro	gp120	C: TV1, 1086	200 µg	MF59	T (210)	ALVAC only: 0, 1; ALVAC + prot/MF59: 3, 6, 12
107 (1-2)	RSA, ZIM, MOZ	06/2017	Canarypox	C: ZM96 gp120; B: gp41^5^, gag, pro	gp120	C: TV1, 1086	200 µg	MF59	T1 (36)	ALVAC only: 0, 1; ALVAC + prot/MF59: 3, 6, 12
107 (1-2)	RSA, ZIM, MOZ	06/2017	Canarypox	C: ZM96 gp120; B: gp41^5^, gag, pro	gp120	C: TV1, 1086	200 µg	Alum	T2 (36)	ALVAC only: 0, 1; ALVAC + prot/alum: 3, 6, 12
107 (1-2)	RSA, ZIM, MOZ	06/2017	Canarypox	C: ZM96 gp120; B: gp41^5^, gag, pro	gp120	C: TV1, 1086	200 µg	MF59	T3 (36)	ALVAC + prot/MF59: 0, 1, 6, 12
107 (1-2)	RSA, ZIM, MOZ	06/2017	Canarypox	C: ZM96 gp120; B: gp41^5^, gag, pro	gp120	C: TV1, 1086	200 µg	None	T4 (24)	ALVAC only: 0, 1; ALVAC + prot/none: 3, 6, 12
120 (1-2)	RSA, TAN, ZAM, ZIM, USA	01/2018	Canarypox	C: ZM96 gp120; B: gp41^5^, gag, pro	gp120	C: TV1, 1086	200 µg	MF59	T1 (100)	ALVAC only: 0, 1; ALVAC+100 µg ea prot/MF59: 3, 6
120 (1-2)	RSA, TAN, ZAM, ZIM, USA	01/2018	Canarypox	C: ZM96 gp120; B: gp41^5^, gag, pro	gp120	C: TV1, 1086	200 µg	AS01_B_	T2 (100)	ALVAC only: 0, 1; ALVAC+100 µg ea prot/AS01_B_: 3, 6
120 (1-2)	RSA, TAN, ZAM, ZIM, USA	01/2018	Canarypox	C: ZM96 gp120; B: gp41^5^, gag, pro	gp120	C: TV1, 1086	40 µg	AS01_B_	T3 (100)	ALVAC only: 0, 1; ALVAC + 20 µg ea prot/AS01_B_: 3, 6
702 (2b-3)	RSA	10/2016	Canarypox	C: ZM96 gp120; B: gp41^5^, gag, pro	gp120	C: TV1, 1086	200 µg	MF59	T (2700)	ALVAC only: 0, 1; ALVAC + prot/MF59: 3, 6, 12, 18
705 (2b)	RSA, MOZ, MAW, ZAM, ZIM	11/2017	Ad26	Mos1: env; Mos2S: env; Mos1.gag-pol; Mos2.gag-pol	gp140	C: C97ZA	250 µg	Alum	T1 (1300)	Ad26 only: 0, 3; Ad26 + prot/alum: 6, 12
096 (1b)	SUI	08/2012	Vaccinia	C: ZM96 gp140, ZM96 gag, CN54 pol-nef	AIDSVAX B/E gp120	B: MN; E: A244	600 µg	Alum	T1 (20)	NYVAC only: 0, 1; NYVAC + prot/alum: 3, 6
096 (1b)	SUI	08/2012	Vaccinia	C: ZM96 gp140, ZM96 gag, CN54 pol-nef	AIDSVAX B/E gp120	B: MN; E: A244	600 µg	Alum	T2 (20)	NYVAC + prot/alum: 0, 1, 3, 6
096 (1b)	SUI	08/2012	DNA	C: ZM96 gp140, ZM96 gag, CN54 pol-nef	AIDSVAX B/E gp120	B: MN; E: A244	600 µg	Alum	T3 (20)	DNA only: 0, 1; NYVAC + prot/alum: 3, 6
096 (1b)	SUI	08/2012	DNA	C: ZM96 gp140, ZM96 gag, CN54 pol-nef	AIDSVAX B/E gp120	B: MN; E: A244	600 µg	Alum	T4 (20)	DNA + prot/alum: 0, 1; NYVAC + prot/alum: 3, 6
106 (1)	USA, SUI	12/2014	DNA; MVA	Nat-B: B 1059 (HV13288); MVA-CMDR: CRF01-AE recomb. gp150 env, gag, pol	None	None	None	None	T1 (30)	DNA Nat-B only: 0, 1, 2; MVA only: 4, 8
106 (1)	USA, SUI	12/2014	DNA; MVA	CON-S: Group M con. HV13287; MVA-CMDR: CRF01-AE recomb. gp150 env, gag, pol	None	None	None	None	T2 (30)	DNA CON-S only: 0, 1, 2; MVA only: 4, 8
106 (1)	USA, SUI	12/2014	DNA; MVA	Mos: HV13284, HV13285, HV13286 env; MVA-CMDR: CRF01-AE recomb. gp150 env, gag, pol	None	None	None	None	T3 (30)	DNA Mosaic only: 0, 1, 2; MVA only: 4, 8
105 (1)	USA	07/2014	DNA	C: ZM96 gp140, ZM96 gag, CN54 pol-nef	AIDSVAX B/E gp120	B: MN, E: A244	600 µg	Alum	T1 (26)	Prot/alum only: 0, 1; DNA only: 3, 6
105 (1)	USA	07/2014	DNA	C: ZM96 gp140, ZM96 gag, CN54 pol-nef	AIDSVAX B/E gp120	B: MN, E: A244	600 µg	Alum	T2 (26)	DNA only: 0, 1; Prot/alum only: 3, 6
105 (1)	USA	07/2014	DNA	C: ZM96 gp140, ZM96 gag, CN54 pol-nef	AIDSVAX B/E gp120	B: MN, E: A244	600 µg	Alum	T3 (26)	DNA only: 0, 1; DNA + prot/alum: 3, 6
105 (1)	USA	07/2014	DNA	C: ZM96 gp140, ZM96 gag, CN54 pol-nef	AIDSVAX B/E gp120	B: MN, E: A244	600 µg	Alum	T4 (26)	DNA + prot/alum: 0, 1, 3, 6
108 (1-2)	USA, RSA	12/2016	DNA	C: ZM96 gp140, ZM96 gag, CN54 pol-nef	gp120	C: TV1, 1086	200 µg	MF59	T1 (30)	DNA only: 0, 1; DNA+100 µg ea prot/MF59: 3, 6
108 (1-2)	USA, RSA	12/2016	DNA	C: ZM96 gp140, ZM96 gag, CN54 pol-nef	gp120	C: TV1, 1086	200 µg	AS01_B_	T2 (50)	DNA only: 0, 1; DNA+100 µg ea prot/AS01_B_: 3, 6
108 (1-2)	USA, RSA	12/2016	DNA	C: ZM96 gp140, ZM96 gag, CN54 pol-nef	gp120	C: TV1, 1086	40 µg	AS01_B_	T3 (50)	DNA only: 0, 1; DNA+20 µg ea prot/AS01_B_: 3, 6
108 (1-2)	USA, RSA	12/2016	DNA	C: ZM96 gp140, ZM96 gag, CN54 pol-nef	gp120	C: TV1, 1086	200 µg	MF59	T4 (30)	DNA+100 µg ea prot/MF59: 0, 1, 6
108 (1-2)	USA, RSA	12/2016	DNA	C: ZM96 gp140, ZM96 gag, CN54 pol-nef	gp120	C: TV1, 1086	200 µg	AS01_B_	T5 (50)	DNA+100 µg ea prot/AS01_B_: 0, 1, 6
108 (1-2)	USA, RSA	12/2016	DNA	C: ZM96 gp140, ZM96 gag, CN54 pol-nef	gp120	C: TV1, 1086	40 µg	AS01_B_	T6 (50)	DNA+20 µg ea prot/AS01_B_: 0, 1, 6
108 (1-2)	USA, RSA	12/2016	DNA	C: ZM96 gp140, ZM96 gag, CN54 pol-nef	gp120	C: TV1, 1086	40 µg	AS01_B_	T7 (50)	20 µg ea prot/AS01_B_: 0, 1, 6
111 (1)	RSA, ZAM, TAN	06/2016	DNA	C: ZM96 gp140, ZM96 gag, CN54 pol-nef	gp120	C: TV1, 1086	200 µg	MF59	T1 (30)	DNA only: 0, 1; DNA + prot/MF59: 3, 6
111 (1)	RSA, ZAM, TAN	06/2016	DNA	C: ZM96 gp140, ZM96 gag, CN54 pol-nef	gp120	C: TV1, 1086	200 µg	MF59	T2 (30)	DNA + prot/MF59: 0, 1, 6
111 (1)	RSA, ZAM, TAN	06/2016	DNA	C: ZM96 gp140, ZM96 gag, CN54 pol-nef	gp120	C: TV1, 1086	200 µg	MF59	T4 (30)	DNA only: 0, 1; DNA*+prot/MF59: 3, 6
111 (1)	RSA, ZAM, TAN	06/2016	DNA	C: ZM96 gp140, ZM96 gag, CN54 pol-nef	gp120	C: TV1, 1086	200 µg	MF59	T5 (30)	DNA*+prot/MF59: 0, 1, 6
124 (1)	USA	03/2018	Polyvalent DNA	A: 92UG037.1 gp120; B: JR-FL gp120; C: 93MW965.26 gp120; CRF01-AE A/E consensus gp120 C: p55 Gag	Polyvalent proteins (gp120)	A: 92UG037.1; B: JR-FL; C: 93MW965.26,				
CRF01-AE A/E consensus	400 µg	GLA-SE	T2 (21)	DNA only: 0, 1, 3; Prot/GLA-SE: 6, 8						
124 (1)	USA	03/2018	Polyvalent DNA	A: 92UG037.1 gp120; B: JR-FL gp120; C: 93MW965.26 gp120; CRF01-AE A/E consensus gp120 C: p55 Gag	Polyvalent proteins (gp120)	A: 92UG037.1; B: JR-FL; C: 93MW965.26; CRF01-AE A/E consensus	400 µg	GLA-SE	T3 (21)	DNA + Prot/GLA-SE: 0, 1, 3, 6, 8

^1^ IOC country codes are used to conserve space. MAW: Malawi; MOZ: Mozambique; RSA: South Africa; SUI: Switzerland; TAN: Tanzania; THA: Thailand; USA: United States of America; ZAM: Zambia; ZIM: Zimbabwe.

^2^ Subtype C ZM96 is based on HIV-1 96ZM651 (accession number AF286224).

^3^ n = Planned sample size as per schema.

^4^ Intramuscular delivery via needle and syringe unless otherwise noted (asterisk denotes DNA by Biojector).

^5^ Only the transmembrane anchor sequence of gp41 was included.

^6^ T consists of T1 and T2 where T1 was given tetanus and T2 was given placebo at day 0.

Canarypox = ALVAC; Vaccinia = NYVAC.

Ad26 = adenovirus 26, con. = consensus, Mos. = Mosaic, pro = protease, prot = protein, recomb. = recombinant.

*DNA by Biojector.

### A polyvalent DNA prime with matched polyvalent protein/GLA-SE boost regimen and B/E ALVAC prime-protein boost regimen showed the highest-ranked IgG and IgG3 bAb responses

**Figure 1.**
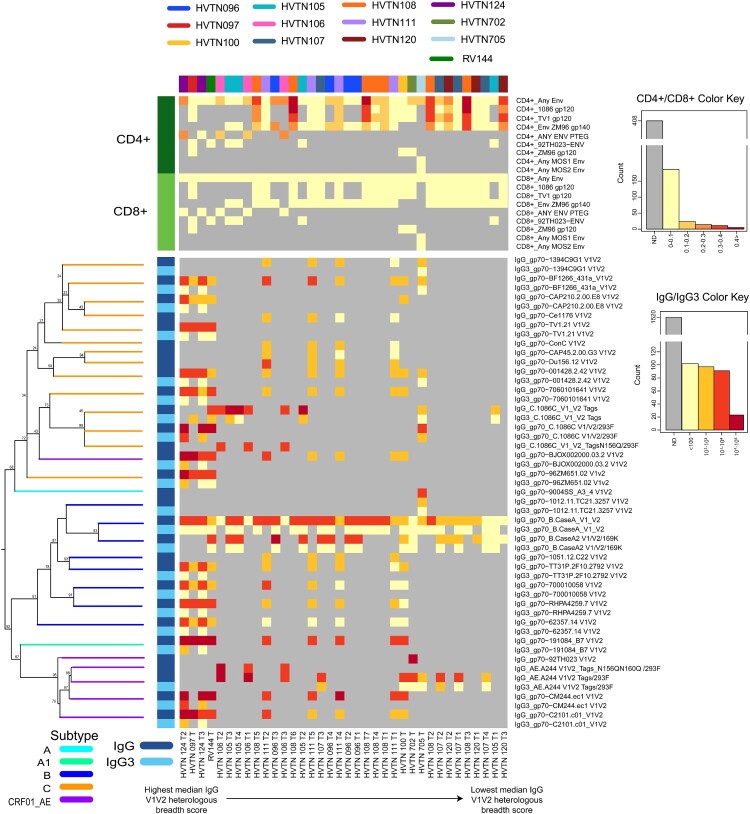
Heatmap of regimen-specific median IgG and IgG3 response magnitudes to heterologous V1V2 antigens and median CD4 + and CD8+ T-cell IFNγ and/or IL-2 expression in response to all measured Env peptide pools. Regimens are ordered horizontally by decreasing median IgG V1V2 heterologous breadth score. Gray cells indicate responses not tested in the corresponding regimen. V1V2 antigens in the IgG/IgG3 panel are ordered vertically by phylogenetic tree of the corresponding V1V2 gp70 sequences (shown on the left with branches color-coded by clade). A total of 32 V1V2 antigens were tested across all regimens, including both the tag and gp70-scaffolded forms of the two antigens 1086 and its N156Q/293F mutated version. Since the two forms have identical sequences, they are represented only once in the phylogenetic tree, for a total of 30 branches. Row bars on the left are color coded by response: dark green for CD4+ T-cells, light green for CD8+ T-cells, dark blue for IgG, and light blue for IgG3. Because some of the V1V2 antigens were tested for IgG only, some of the tree branches point to a single row, while others correspond to two rows in the heatmap (antigens for which both IgG and IgG3 responses were tested).

**Figure 2.**
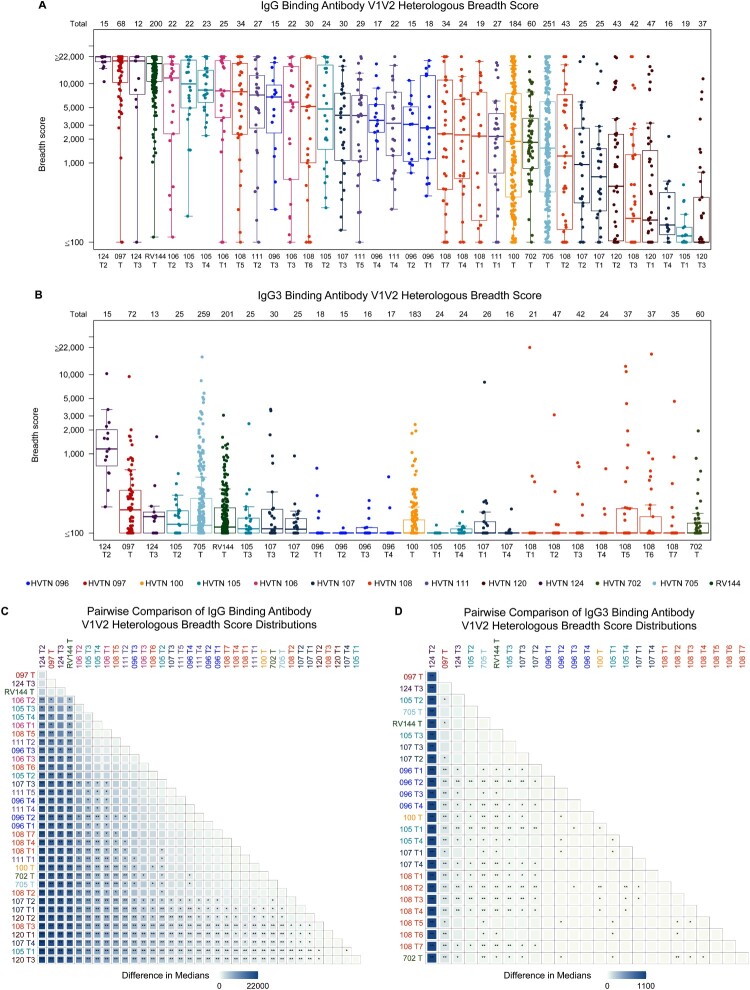
(A, B) Distributions of binding antibody V1V2 heterologous breadth scores in each regimen and (C, D) pairwise comparisons between regimens of binding antibody V1V2 heterologous breadth score distributions. The box plots show the distribution of (A) IgG and (B) IgG3 binding antibody V1V2 heterologous breadth scores across participants in a given regimen, where each dot represents one participant. The horizontal line in each box represents the median regimen-specific breadth score. The number of participants (n) in each regimen is provided in the top row. Within a given regimen, the V1V2 heterologous breadth score was defined as the geometric mean of the binding antibody responses to the 3 heterologous V1V2 antigens with the highest median responses among all participants in that regimen. The tile plots display the difference in medians for each regimen pair in the (C) IgG and (D) IgG3 binding antibody V1V2 heterologous breadth score with asterisks denoting statistical significance: ** Wilcoxon test *p*-value < 0.001; * Wilcoxon test *p*-value < 0.05.

IgG and IgG3 bAb responses were compared across trials based on their breadth scores, to account for the differing number of antigens assessed in each trial (Tables S1, S2). The breadth score for a given isotype was defined as the geometric mean of the bAb responses to the 3 heterologous V1V2 antigens with the highest median responses among all participants by trial and vaccine group. The highest median IgG and IgG3 breadth scores were seen with the HVTN 124 prime-boost or co-administered polyvalent DNA with matched polyvalent protein/GLA-SE regimens and with the B/E ALVAC prime-boost regimen of HVTN 097 (Figures 1 and 2A, B). No significant differences among IgG breadth scores were observed among these three regimens. The HVTN 124 prime-boost polyvalent DNA with matched polyvalent protein/GLA-SE regimen and the HVTN 097 and RV144 B/E ALVAC prime-boost regimens had significantly higher scores than all other regimens (Figure 2C). Moreover, when considering IgG breadth scores based on clade B and C antigens only, the HVTN 124 prime-boost polyvalent DNA with matched polyvalent protein/GLA-SE regimen had significantly higher scores than all other regimens apart from HVTN 105 T3 (Figure S2A, S2C). In addition, the HVTN 124 prime-boost polyvalent DNA with matched polyvalent protein/GLA-SE regimen induced IgG3 overall and clade B + C breadth scores of significantly greater magnitude than all other regimens, including HVTN 097, RV144, and HVTN 105 T3 (Figure 2D; Figure S2B, S2D, all *p* < 0.001). IgG and IgG3 responses to Con 6 gp120/B were also very robust (Figures S7, S8).

### The prime-boost matched polyvalent DNA and polyvalent protein/GLAS-SE regimen in HVTN 124 induced broad and strong bAbs and robust CD4+ T-cell responses

The HVTN 124 prime-boost regimen of polyvalent DNA with matched polyvalent protein/GLA-SE induced strong IgG and IgG3 bAb responses to heterologous Env antigen sequences across all tested subtypes (A1, B, C, and CRF01_AE) (Figure 1). The IgG medians of the prime-boost HVTN 124, co-administered HVTN 124 and HVTN 097 regimens exceeded 10,000 MFI for 6/16 (37.5%), 3/16 (18.8%) and 3/14 (21.4%) of the heterologous V1V2 antigens evaluated (Figure 1). Within HVTN 124, the prime-boost regimen induced significantly stronger IgG3 breadth scores (Figure 2, *p* < 0.001), IgG3 response rates and magnitudes to Con 6 gp120/B (Figure S8, p’s < 0.001), and higher CD4+ T cell response rate (100%, Figure 4A) and magnitudes (Figure 4B) to Env than the co-administered regimen (Figure 4, *p* = 0.005); neither regimen induced a CD8+ T cell response to Env (Figure S5). Notably, the CD4+ T-cell responses in HVTN 124 were to global potential T cell epitope peptide pools for Env [35], not to vaccine-matched peptide pools as were assessed in most other trials (Table S4). IgG breadth scores exceeded 10,000 netMFI for all participants receiving the prime-boost regimen (Figure 2A) and median IgG3 breadth score was > 5.9-fold higher than all other regimens (Figure 2B). IgG3 magnitudes to Con 6 gp120/B were also significantly higher than most (19/25) other regimens (Figure S8).

### B/E ALVAC + protein/alum prime-boost regimen induced strong bAbs but weak T-cell responses

**Figure 3.**
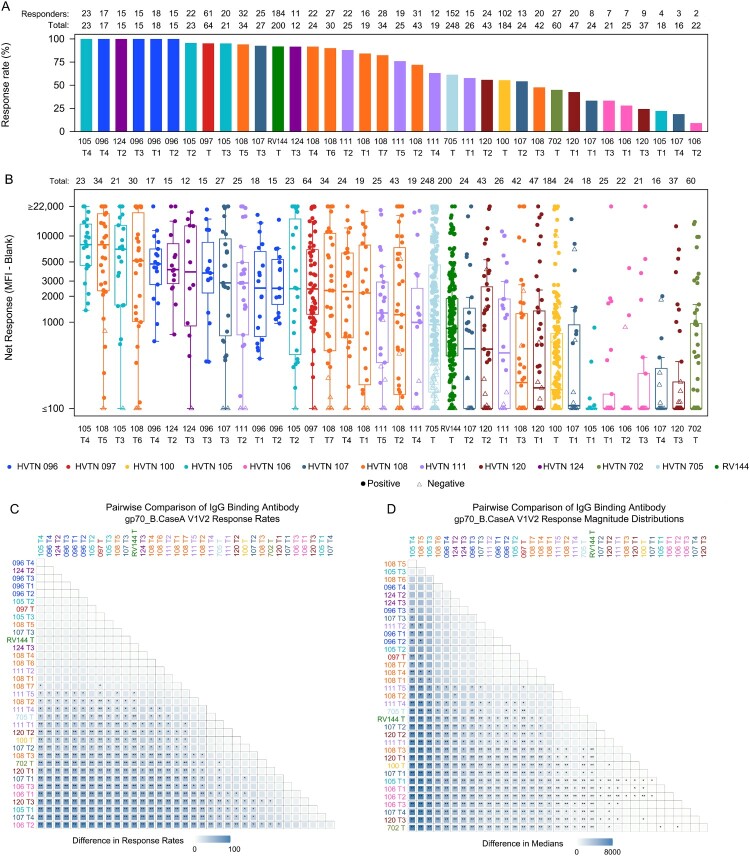
IgG binding antibody responses to gp70_B.CaseA V1V2 by trial and comparisons between arms across trials: (A) response rates, (B) response magnitude distributions, (C) pairwise comparison between regimens of response rates, (D) pairwise comparison between regimens of response magnitude distributions. The box plot in (B) shows the distribution of IgG binding antibody response magnitudes to gp70_B.CaseA V1V2 across participants in a given regimen, where each dot represents one participant. The horizontal line in each box represents the median regimen-specific IgG gp70_B.CaseA V1V2 response magnitude. The number of participants (n) in each regimen is provided in the top row (Total). The tile plots in (C) and (D) display the difference for each regimen pair in (C) response rate and (D) median response magnitude, with asterisks denoting statistical significance. In (C): ** indicates Barnard’s test *p*-value < 0.001, * indicates *p*-value < 0.05 (D) magnitude comparison between arms across trials. In (D): ** indicates Wilcoxon test *p*-value < 0.001, * indicates *p*-value < 0.05.

**Figure 4.**
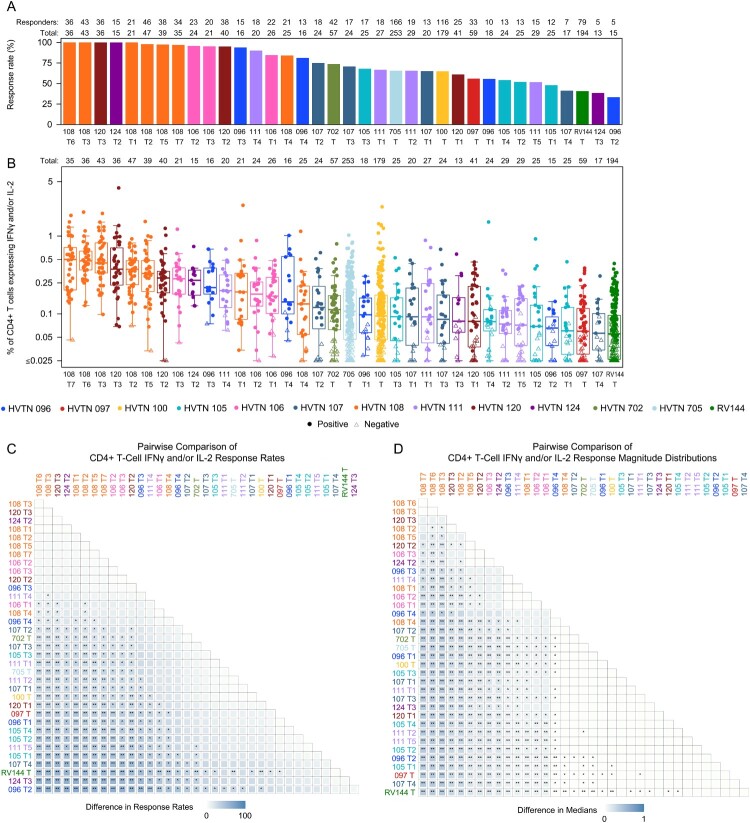
CD4+ T cell IFN-γ and/or IL-2 expression in response to Any Env by trial: (A) response rates, (B) response magnitudes, (C) pairwise comparison between regimens of response rates, (D) pairwise comparison between regimens of response magnitude distributions. The box plot in (B) shows the distribution of % CD4+ T cells expressing IFN-γ and/or IL-2 in response to Any Env across participants in a given regimen, where each dot represents one participant. The horizontal line in each box represents the median regimen-specific % CD4+ T cells expressing IFN-γ and/or IL-2 in response to Any Env. The number of participants (n) in each regimen is provided in the top row (Total). The tile plots in (C) and (D) display the difference for each regimen pair in (C) response rate and (D) median response magnitude, with asterisks denoting statistical significance. In (C): ** indicates Barnard’s test *p*-value < 0.001, * indicates *p*-value < 0.05. In (D): ** indicates Wilcoxon test *p*-value < 0.001, * indicates *p*-value < 0.05.

In addition to the polyvalent DNA with polyvalent protein/GLA-SE regimens of HVTN 124, strong IgG bAb responses to heterologous V1V2 Env antigens were also seen with the ALVAC + gp120 protein/alum regimen evaluated in HVTN 097 and RV144 (Figure 1). Fewer antigens were assessed in these trials than in HVTN 124, particularly for IgG3 in HVTN 097, however the antigens represented all 4 subtypes of interest: A1, B, C, and CRF01_AE. The HVTN 097 regimen and both HVTN 124 regimens generated greater than 2.8-fold higher median IgG magnitudes than RV144 to the gp70_B.CaseA V1V2 antigen with greater than 2.6-fold median magnitudes than the two recent efficacy trials HVTN 702 and 705; high IgG responses to this antigen were associated with reduced acquisition risk in RV144 [2] (Figure 3B, D). RV144 and HVTN 097 both induced lower CD4+ T cell response rates and magnitudes to Env than most other trials, including the prime-boost regimen in HVTN 124 (Figure 4A, C, all *p* ≤ 0.003). Positive CD8+ T-cell responses to Any Env were observed in 0–5% of participants in all regimens in each of the three trials (Figure S5).

### DNA + MVA prime-boost regimens induced moderate bAb and CD4+ T-cell responses

The IgG breadth scores of the DNA + MVA regimens evaluated in HVTN 106 T2 and T1 were significantly lower than those of the prime-boost regimen of polyvalent DNA with matched polyvalent protein/GLA-SE (HVTN 124 T2) and the ALVAC + protein/alum regimen (HVTN 097, RV144) (Figure 2C; all *p* < 0.05). Nevertheless, the HVTN 106 T2 and T1 DNA + MVA regimens stimulated relatively strong IgG responses to 3 of the 4 heterologous antigens tested, with GMs > 1,000 MFI (Figure 1). The median IgG magnitudes to gp70_B.CaseA V1V2 and Con 6 gp120/B for these groups, however, were among the lowest across all trials (Figure 3, Figure S7). Despite assessing few heterologous antigens for IgG and none for IgG3 in HVTN 106, the antigens measured for IgG covered subtypes B, C, and CRF01_AE (Figure 1). CD4+ T-cell responses to Any Env were comparable to those seen in the prime-boost regimen of polyvalent DNA with matched polyvalent protein/GLA-SE in HVTN 124 (Figure 4), while also inducing the highest Env-specific CD8+ T cell response rate across trials (54%) vs. 0% in the HVTN 124 regimens (Figure S5).

### Other DNA + protein/adjuvant regimens induced moderate bAb but weak CD4+ T-cell responses

The DNA prime-DNA boost and DNA co-administered regimens with protein/alum evaluated in HVTN 105 (T3, T4) induced moderately strong IgG heterologous breadth scores, with medians of 9974.5 and 8319.0 MFI, respectively. Although these were significantly lower than those seen in the prime-boost regimens of HVTN 124, HVTN 097 and RV144 (Figure 2C, all *p* < 0.05), IgG geometric means to 2 of the 3 V1V2 antigens were 10-fold higher for the DNA prime-DNA boost and co-administered regimens of HVTN 105 than the ALVAC regimen of RV144 (Figure 1). Of note, the HVTN 105 breadth scores are comprised of responses to the subtype C 1086 and subtype B gp70_B.CaseA V1V2 antigens (each identified as correlates of risk in RV144 [2]) as no other heterologous antigens were assessed. The magnitudes of IgG bAb responses to gp70_B.CaseA V1V2 were significantly higher for HVTN 105 T3 and T4 than for RV144 (Figure 3B, D, both *p* < 0.001) with significantly higher IgG magnitudes to Con 6 gp120/B for HVTN 105 T3 compared to RV144 (Figure S7, *p* = 0.008). Both the prime-boost and co-administered regimens of HVTN 105 induced significantly lower Env-specific CD4 + and CD8+ T-cell responses than most other trials (Figure 4 and Figure S5; *p* = 0.04 and *p* = 0.02, respectively).

### AS01_B_ adjuvant enhanced cellular and bAb responses compared to MF59 adjuvant

The AS01_B_-adjuvanted vaccine regimens of HVTN 120 T2, HVTN 108 T2 and T3 with the highest median CD4+ T-cell responses to Any Env tended to have lower IgG and IgG3 responses to heterologous V1V2 antigens (Figure 1) with robust IgG responses to Con 6 gp120/B (Figure S7). The AS01_B_-adjuvanted regimens of HVTN 108 induced high magnitude CD4+ T-cell responses to Any Env with median magnitudes exceeding 0.37% of CD4+ T cells expressing IFN-γ and/or IL-2 (Figure 4) as well as moderately strong IgG and IgG3 responses to gp70_B.CaseA V1V2 (Figure 3, Figure S4) but lower IgG and IgG3 breadth scores with only one other heterologous antigen assessed (Tables S1, S2). The DNA AS01_B_-adjuvanted groups of HVTN 108 (T2, T3, T5, T6, T7) included the same protein inserts as the MF59-adjuvanted regimens of HVTN 100, HVTN 107 and HVTN 702, yet had significantly higher CD4+ T cell response magnitudes to Env (Figure 4B, all *p* < 0.05). The ALVAC prime, AS01_B_-adjuvanted protein boost regimen of HVTN 120 induced also high CD4+ T-cell responses to Any Env yet had very low IgG breadth scores as well as low IgG responses to gp70_B.CaseA V1V2; IgG3 responses were not measured. Similarly, the higher dose protein with MF59 (the same regimen assessed in HVTN 100 and 702) or with AS01_B_ adjuvant in HVTN 120 (T1, T2, respectively) induced low IgG breadth scores (median magnitudes < 498.0) and low responses to gp70_B.CaseA V1V2 (median magnitudes < 466.3) (Figures 2A & C and 3). Significantly higher CD4+ T cell response magnitudes to Env were seen for the low protein dose in HVTN 120 T3 compared to the MF59-adjuvanted and the higher protein dose AS01_B_ regimens in HVTN 120 T1 and T2 (Figure 4, both *p* < 0.05).

### Protein/alum followed by DNA induced low bAb and cellular responses

The second lowest ranked median IgG breadth score and fourth lowest ranked median CD4+ T-cell responses to Any Env were observed with the protein/alum + DNA regimen of HVTN 105 (T1). This regimen included similar inserts as RV144, HVTN 100, HVTN 107, and HVTN 702 but delivered adjuvanted protein first, at months 0 and 1 rather than at months 3, 6, 12. The HVTN 105 regimen contained the same A244 protein insert as RV144 and the full length gp140 Env ZM96 insert for DNA, similar to the gp120 ZM96 insert used in the ALVAC for HVTN 100, HVTN 107, and HVTN 702. The ALVAC prime-boost regimens of HVTN 107 (unadjuvanted or adjuvanted with MF59 or alum), HVTN 100, and HVTN 702 and the mosaic Ad26 with protein/alum in HVTN 705 also all induced low IgG breadth scores although interestingly, HVTN 705 induced moderate IgG3 breadth scores compared to the other regimens studied (Figure 2).

### Robust cellular responses to Gag seen only with DNA + NYVAC protein/alum regimens with weak CD4+ Env and bAb responses

The only regimens generating a considerable CD4 + and CD8+ T cell response to any Gag, with medians exceeding 0.10 for CD4+, were the DNA + NYVAC + protein/alum regimens of HVTN 096 (Figures S3, S6). These regimens were not among the top ranked regimens with respect to CD4+ T-cell responses to Any Env, IgG V1V2 breadth score, or IgG3 V1V2 breadth score (Figures 4, 2).

### Global diversity of antigen panel used for bAb assessment and vaccine inserts of the 36 regimens

The analyses above were focused on comparing immunogenicity across the vaccine regimens. Given that these regimens tested represented a diversity of subtypes, we concluded by performing a global analysis of the regimen immunogens to assess how well the different vaccine inserts and V1V2 antigens used in bAb assessment represented the viral diversity of circulating strains, approximated by downloading env sequences from the LANL database (details in Supplementary Methods). The antigens were part of a larger panel of 43 gp70 antigens [36] and represented subtypes C (n = 13), B (n = 8), A (n = 1), A1 (n = 1), and circulating recombinants CRF01_AE (n = 5) and CRF07_BC (n = 1) (Figure S9). Five out of 12 vaccine inserts were subtype C, 1 clade A, 1 clade B, 2 clade CRF01_AE and 3 mosaics (Figure 1, Table 1, Figure S9). The predominance of subtype C and, to a lesser extent, B in the antigens and inserts in the tested regimens tracked with the geographic locations where the regimens were tested (Table 1).

Subtype C and CRF01_AE antigens and vaccine inserts were equally distributed within their own clades in the global midpoint-rooted phylogenetic tree (Figure S10), suggesting that the assayed subtype C and CRF01_AE antigens well-represented the viral diversity of circulating subtype C and CRF01_AE strains. In contrast, subtype B antigens and vaccine inserts were mostly concentrated in the B subclade closer to the tree root, suggesting that the assayed subtype B antigens represented global subtype B diversity less well. To more closely examine these results, Figure S11 shows V1V2 branch length of past circulating viruses (plotted by sampling year) as well as the branch lengths of the 30 gp70 V1V2 antigen sequences. Viruses sampled between 2010 and 2021 generally exhibited higher diversity than the 13 subtype C antigens included in this panel in the core epitope of the V2 domain (HXB2 positions 169-171), where key contacts for V2 bnAbs are often found. Figure S12 additionally compares V1V2 variable characteristics of the 30 antigen sequences to those of past circulating viruses, with the antigens having higher median V1V2 length (72 vs. 70 amino acids) and a significantly higher (*p* = 0.0016 by Wilcoxon test) median net charge (0 vs. -1.5) (Figure S12). Negative V1V2 net charge has been associated with higher resistance to V2 bnAbs such as PG9, PGT145, PGDM1400, and CAP256-VRC26 [37]. A potential limitation of this analysis is that while subtype C is globally the most represented clade (estimated at ∼47% of all global acquisitions [38,39]) over half of the downloaded sequences (2,864, or 60%) were clade B, due to an over-representation in the database (Figure S10).

## Discussion

The polyvalent DNA prime with matched polyvalent protein/GLA-SE boost regimen (HVTN 124) and the B/E ALVAC prime-boost regimen (HVTN 097, RV144) induced IgG breadth scores superior to those of all other regimens, with the same HVTN 124 prime-boost regimen also having a clade B + C breadth score superior to all but the DNA + AIDSVAX/alum prime-boost regimen in HVTN 105 T3. The HVTN 124 prime-boost regimen also induced significantly higher IgG3 overall and clade B + C breadth scores than all other regimens along with strong CD4+ T-cell responses. Notably, the HVTN 124 regimen’s IgG3 breadth scores were markedly higher than those of HVTN 097 and RV144, the only regimen to have demonstrated modest vaccine efficacy, and the HVTN 124 regimen’s IgG overall breadth score was very similar to those of HVTN 097 and RV144. A distinctive vaccine design aspect of HVTN 124 study was the use of matched polyvalent Env inserts between the DNA prime and protein boost. Given the use of these polyvalent inserts, a unique study design aspect of the HVTN 124 and HVTN 106 trials was the assessment of CD4+ T-cell responses to global PTE Env peptide pools, instead of vaccine-matched peptide pools. The global pools represent a common, standardized panel of HIV-1 peptides relevant to vaccine evaluation, with uniform coverage across the major subtypes A, B, C [35]. Therefore, the robust CD4+ T-cell responses elicited by HVTN 124 are impressive. Moreover, the HVTN 124 regimen elicited strong, broad IgG and IgG3 Env V1V2-directed bAb responses. These results cumulatively suggest that DNA is a good prime when followed by matched protein antigens boost, capable of inducing better binding and cellular immune responses than those observed in the two most recent vaccine efficacy trials, HVTN 702 and 705. Additional clinical trials would be needed to validate the findings reported in the current study in other study populations.

The HVTN 124 trial was also unique in its use of the GLA-SE adjuvant. While our data do not allow any direct comparison of adjuvant performance in the polyvalent DNA–matched polyvalent protein/GLA-SE prime-boost regimen, robust CD4+ T-cell responses and strong, broad IgG and IgG3 V1V2-directed bAb responses were seen with the use of GLA-SE adjuvant with the protein boost. Thus, this adjuvant may merit further exploration in future.

Our study was the first to include such a large number of HIV vaccine clinical trials (13) and regimens (36) with a focus on two key immune correlates of protection. The trials used standardized designs, case report forms, and harmonized immunogenicity endpoints, with the specimens analyzed here collected at a common time point [2 weeks (4 weeks for HVTN 705) after the final primary series HIV immunization], and assayed in centralized laboratories using standardized, validated assays. A single Statistical and Data Management Center was responsible for all data cleaning and analysis.

Our study also has limitations. As discussed above, the majority of trials (HVTN 124 and HVTN 106 being the two exceptions) included in this analysis assessed T-cell responses to vaccine-matched peptide pools, and thus the T-cell responses to heterologous peptide pools are unknown. The responses to vaccine-matched antigens are expected to be higher than those to the global PTEs measured in HVTN 124 and HVTN 106. Moreover, the peptide pool set and size (1-5 peptides) for T-cell response assessment differed by trial, as well as the antigen set and size (2-16 antigens) for bAb response assessment. Further harmonization through the use of a standard antigen panel would have strengthened the analysis through more direct antigen comparisons. An additional limitation of this cross-protocol analysis was that it only included immune responses that were measured in all of the trials, on which we expand below.

Serum IgA binding antibodies against Env were not measured in all of the trials and thus were not included in the present analysis. These antibodies were identified as a direct correlate of risk in RV144 vaccine recipients, with interaction analyses showing that IgG avidity, antibody-dependent cellular cytotoxicity, neutralizing antibodies, and CD4+ T cells were borderline-significant inverse correlates of risk in vaccine recipients with low (but not high) Env-specific IgA antibodies [2]. Follow-up studies suggested that vaccine-elicited IgA antibodies may block and interfere with IgG mediated ADCC [40,41]. The HVTN 124 prime-boost regimen elicited low serum IgA response rates (ranging from 13% to 53%) and geometric mean serum IgA response magnitudes ranging from 2697 to 22026 to four autologous gp120 antigens, and also elicited ADCC activity [28]. It is unknown whether serum IgA antibodies elicited by this regimen similarly inhibit ADCC activity.

The current focus of the HIV vaccine field has moved towards pursuing HIV-1 vaccines that induce broadly neutralizing antibodies (bnAbs) [42,43], under the well-supported hypothesis that achieving high levels of broad, highly potent bnAbs is central (or perhaps even necessary) for achieving protection from the extensive genetic diversity of circulating HIV-1 strains. If this important goal can be achieved, the tremendous capacity of HIV-1 to evade the host humoral immune response and the potential emergence of bnAb-resistant variants will remain a point of consideration. In addition, while important steps have been made in the development of HIV vaccines designed to induce bnAbs in humans [44,45], progress has been slow due to the many challenges in this approach [46]. Passive infusion is another potential approach for HIV prevention via bnAbs that circumvents some of these challenges, however, the same hurdles of HIV-1 diversity and antibody resistance still apply. The AMP trials tested the prevention efficacy of the passively infused broadly neutralizing monoclonal antibody (mAb) VRC01 at two doses vs. placebo, and showed that VRC01 only prevented acquisition of HIV-1 viral isolates that were sensitive to VRC01 [47]. This finding highlighted the need to target multiple mAb epitopes via combination mAb regimens and/or multi-specific (bi- or tri-) antibodies [48–51].

Synthesis of the HIV-1 immune correlates results to date has suggested that there may be multiple types of protective immune responses, which could in act in concert or sequentially, supporting the idea of a “complex correlate” [52,53]. Thus, in addition to pursuing bnAb-based approaches, it may be advantageous for the field to additionally pursue complementary, multifunctional strategies such as vaccines that can induce polyfunctional antibody responses (neutralizing as well as non-neutralizing, e.g. ADCC and/or antibody-dependent phagocytosis [54]) – as well as potent and broadly cross-reactive T-cell responses – as an integrated strategy may ultimately be required [55]. In this light, the HVTN 124 prime-boost regimen did elicit a promising combination of neutralizing antibody, non-neutralizing antibody (ADCC), and cellular responses [28]. Our comparative analysis further confirmed the strength of the DNA prime-protein boost approach with matched polyvalent Env antigens relative to other recent major HIV vaccine clinical trials with respect to potent V1V2 antibody breadth and strong CD4+ T-cell responses.

### HVTN 096

The HVTN 096 trial was additionally supported by NIAID PHS grant UM1 AI069481. The DNA vaccine was provided by the IPPOX Foundation in Switzerland through support from the Collaboration of AIDS Vaccine Discovery of the Bill & Melinda Gates Foundation (award OPP52845), NYVAC vaccine was provided by the EuroVacc Foundation in Switzerland, and Env protein (AIDSVAX B/E) was provided by Global Solutions for Infectious Diseases and the US Military HIV Research Program.

### HVTN 097

The HVTN 097 trial was additionally supported by NIAID grants UM1 AI069453 [Soweto-Bara Clinical Research Site], UM1 AI069519, and UM1 AI069469. The James B. Pendleton Charitable Trust donated equipment, additional funding for laboratory assays was provided by the South African Medical Research Council, and the Bill & Melinda Gates Foundation contributed to the Cape Town HVTN Immunology Laboratory facility.

### HVTN 100

The HVTN 100 trial was additionally supported by NIAID grants UM1 AI069453 [Soweto-Bara Clinical Research Site], UM1 AI069519, UM1 AI069469, and UM1 AI069422, and The Bill & Melinda Gates Foundation award OPP1110792. The Bill & Melinda Gates Foundation provided funding to Fred Hutchinson Cancer Research Center to support the implementation of HVTN 100 at the Soweto-Bara clinical research site, Cape Town – Emavundleni clinical research site, Durban – eThekwini clinical research site, Durban – Isipingo clinical research site, and the Klerksdorp and Soshanguve sites. Funding was provided to Novartis Vaccines and Diagnostics (now part of GSK) by NIAID (HHSN272201300033C//HHSN272201600012C) for selection and process development of the two gp120 envelope proteins, TV1.C and 1086.C, and by the Bill & Melinda Gates Foundation Global Health Grant OPP1017604 and NIAID for the manufacture and release of the gp120 clinical grade material.

### HVTN 105

The HVTN 105 trial was additionally supported by NIAID grants UM1 AI069511, UM1 AI069470, UM1 AI069534, P30 AI450008, UM1 AI069439, UM1 AI069481, and UM1 AI069496. Additional support was also provided by the National Center for Advancing Translational Sciences, NIH, through grant number UL1TR001873 (Columbia). The DNA vaccine was provided by the IPPOX Foundation, Switzerland, through support from the Collaboration of AIDS Vaccine Discovery of the Bill & Melinda Gates Foundation grant OPP52845.

### HVTN 106

The HVTN 106 trial was additionally supported by NIAID grants UM1 AI069412 and UL1 RR025758 to Harvard, P30 AI064518 to Duke Center for AIDS Research, UM1 AI144371 to Consortia for HIV/AIDS Vaccine Development, and UM1 AI100645 to Center for HIV/AIDS (Vaccine Immunology–Immunogen Design), as well as award OPP52282 from the Bill & Melinda Gates Foundation.

### HVTN 107

The HVTN 107 trial was additionally supported by NIAID grants AI069501, P30 AI064518, [Duke CFAR], and UM1 AI069453 [Soweto-Bara Clinical Research Site]. Funding was provided to Novartis Vaccines and Diagnostics (now part of GSK) by NIH (HHSN272201600012C) for the production process development of two gp120 envelope proteins TV1.C and 1086.C, and by the Bill & Melinda Gates Foundation Global Health (Grant OPP1017604) for the manufacture and release of the gp120s clinical grade material.

### HVTN 108

The HVTN 108 trial was additionally supported by the Bill and Melinda Gates Foundation. Funding was provided to Novartis Vaccines and Diagnostics (now part of GSK) by NIAID (HHSN272201300033C//HHSN272201600012C) for the selection and process development of the two gp120 envelope proteins TV1.C and 1086.C, and by the Bill & Melinda Gates Foundation Global Health Grant OPP1017604 and NIAID for the manufacture and release of the gp120 clinical grade material.

### HVTN 111

The HVTN 111 trial was additionally supported by NIAID grants UM1 AI069453, UM1 AI069422, UM1 AI108568, and UM1 AI069423; grants HHSN272201300033C/HHSN272201600012C for development of the glycoprotein [gp] 120 envelope proteins TV1.C and 1086.C and support for the manufacture and release of the gp120 clinical grade material to Novartis Vaccines and Diagnostics (now part of GSK); the Bill & Melinda Gates Foundation (OPP1110830; funding to the Fred Hutchinson Cancer Research Center to support the implementation of HVTN 111 at the Klerksdorp Clinical Research Site, Lusaka-Matero Clinical Research Site, and Tembisa Clinical Research Site; and global health grant OPP1017604 to Novartis Vaccines and Diagnostics [now part of GSK]). The DNA vaccine was provided by the IPPOX Foundation, Switzerland, through support from the Collaboration of AIDS Vaccine Discovery of the Bill & Melinda Gates Foundation (grant OPP52845).

### HVTN 120

The HVTN 120 trial was additionally supported by NIAID grants UM1 AI069511 and UM1 AI069453 [Soweto-Bara Clinical Research Site]. Funding was provided to Novartis Vaccines and Diagnostics (now part of GSK) by NIAID (HHSN272201300033C//HHSN272201600012C) for the selection and process development of the two gp120 envelope proteins TV1.C and 1086.C, and by the Bill & Melinda Gates Foundation Global Health Grant OPP1017604 and NIAID for the manufacture and release of the gp120 clinical grade material.

### HVTN 124

The HVTN 124 trial was additionally supported by NIAID IPCAVD U19AI082676 (UMass Medical School/Waisman Biomanufacturing for vaccine manufacture), NIAID P30 AI064518 (Duke Center for AIDS Research), NIAID UM1 AI069534 (University of Pennsylvania HIV Clinical Trials Unit), NIAID P30 AI045008 (Penn Center for AIDS Research), and NIAID UM1 AI069511 (University of Rochester HIV/AIDS Clinical Trials Unit). The Access to Advanced Health Institute (AAHI) provided the GLA-SE adjuvant formulation.

### HVTN 702

The HVTN 702 trial was additionally supported by grants (HHSN272201300033C and HHSN272201600012C) to Novartis Vaccines and Diagnostics (now part of GSK) by the National Institute of Allergy and Infectious Diseases (NIAID) of the National Institutes of Health (NIH) for the selection and process development of the two gp120 envelope proteins TV1.C and 1086.C and by the Bill and Melinda Gates Foundation Global Health Grant (OPP1017604) and NIAID for the manufacture and release of the gp120 clinical grade material. GSK contributed financially to the provision of preexposure prophylaxis to trial participants. The South African Medical Research Council supported its affiliated research sites.

### HVTN 705

The HVTN 705 trial was additionally supported by Janssen Vaccines & Prevention BV; Bill & Melinda Gates Foundation; Ragon Institute of the Massachusetts Institute of Technology, Massachusetts General Hospital, and Harvard; and US Army Medical Materiel Development Activity. Under the grant conditions of the Foundation, a Creative Commons Attribution 4.0 Generic License has already been assigned to the Author Accepted Manuscript version that might arise from this submission.

### RV144

The RV144 trial was supported in part by an Interagency Agreement (Y1-AI-2642-12) between the U.S. Army Medical Research and Materiel Command and the National Institute of Allergy and Infectious Diseases and by a cooperative agreement (W81XWH-07-2-0067) between the Henry M. Jackson Foundation for the Advancement of Military Medicine and the U.S. Department of Defense. Sanofi Pasteur provided the ALVAC-HIV vaccine, and Global Solutions for Infectious Diseases (VaxGen) provided the reagents for the immunogenicity assays.

### Role of the funding source

#### HVTN 096

The funder contributed to, reviewed, and approved the study's design, data analysis, and preparation of the report and concurred with the decision to submit for publication. The funder had no role in data collection or statistical analyses.

#### HVTN 097

Within the terms of the Grant Award of the Cooperative Agreement with the HVTN, NIAID, as protocol sponsor, contributed to, reviewed, and approved the HVTN 097 study design. NIAID contributed to review and analysis of data, and preparation of the manuscript, and concurred with the decision to submit for publication, but was not involved in the data collection and did not perform statistical analyses.

#### HVTN 100

Within the terms of the grant award of the cooperative agreement with the HVTN, NIAID – as protocol sponsor – contributed to, reviewed, and approved the HVTN 100 study design, established the prespecified immunological criteria for advancing this regimen, and reviewed data from HVTN 100 against those criteria. Along with other members of the Pox-Protein Public-Private Partnership (P5), The Bill & Melinda Gates Foundation also contributed to, reviewed, and approved the HVTN 100 study design, established the prespecified immunological criteria for advancing this regimen, and reviewed data from HVTN 100 against those criteria. The funders contributed to, reviewed, and approved the HVTN 100 study design, established the prespecified immunological criteria for advancing this regimen, and reviewed data from HVTN 100 against those criteria.

#### HVTN 105, 106, 107, 108, 111, 120, 124, 702, 705

Within the terms of the Grant Award of the Cooperative Agreement with the HVTN, NIAID, as protocol sponsor, contributed to, reviewed, and approved the study design. NIAID contributed to review and analysis of data, and preparation of the manuscript, and concurred with the decision to submit for publication, but was not involved in the data collection and did not perform statistical analyses.

#### RV144

ALVAC-HIV (vCP1521) and ALVAC placebo were supplied by the manufacturer, Sanofi Pasteur. AIDSVAX and AIDSVAX placebo (VaxGen) were purchased by the Division of AIDS, National Institute of Allergy and Infectious Diseases, for the purpose of the trial. The US Army Medical Research and Materiel Command participated fully in data collection, determination of the analysis plan, and interpretation of data. The National Institute of Allergy and Infectious Diseases also participated in analysis and data interpretation.

The content is solely the responsibility of the authors and does not necessarily represent the official views of the National Institutes of Health, the National Institute of Allergy and Infectious Diseases, the Department of Health and Human Services, the Centers for Disease Control and Prevention, or The Bill & Melinda Gates Foundation. Material has been reviewed by the Walter Reed Army Institute of Research. There is no objection to its presentation and/or publication. The opinions or assertions contained herein are the private views of the author, and are not to be construed as official, or as reflecting true views of the Department of the Army, the Department of Defense or HJF. The investigators have adhered to the policies for protection of human participants as prescribed in AR 70-25.

## Trademark statement

MF59 is a trademark of Novartis. AS01_B_ is a liposome-based Adjuvant System containing 3-O-desacyl-4'-monophosphoryl lipid A (MPL) (50 mg) and QS-21 (50 µg). AS01_B_ is a trademark owned by or licensed to GSK.

## Supplementary Material

XP_13trials_Moodie_Appendix_EmergMicrobesInf_12Mar2025_clean.docx

## Data sharing

Data and protocols for HVTN 096, 100, 105, 111, 120, and 702 are publicly available online at https://atlas.scharp.org/cpas/project/HVTN%20Public%20Data/begin.view. Data and protocols for HVTN 097, 106, 107, 108, and 124 will also be made publicly available at the same website as above. Data for HVTN 705 may be obtained upon publication from the authors upon reasonable request as determined by an agreement with Yale Open Data Access [YODA] Project through the site http://yoda.yale.edu.

For the RV144 trial, deidentified participant-level data and accompanying research resources are available upon request to communications@hivresearch.org. Distribution of data will require compliance with all applicable regulatory and ethical processes.
