# Supplementary material for: A polyvalent DNA prime with matched polyvalent protein/GLA-SE boost regimen elicited the most robust and broad IgG and IgG3 V1V2 binding antibody and CD4+ T cell responses among 13 HIV vaccine trials
Source: Emerg Microbes Infect. 2025 Apr 7;14(1):2485317. doi: 10.1080/22221751.2025.2485317 (PMC11980190; doi:10.1080/22221751.2025.2485317)
Supplement: XP_13trials_Moodie_Appendix_EmergMicrobesInf_12Mar2025_clean.docx [file TEMI_A_2485317_SM0124.docx]

**Supplementary Methods and Results**

**Binding Antibody Multiplex Assay (BAMA)**

The positive controls consisted of purified polyclonal IgG from people with HIV (HIVIG NIH AIDS Reagent Program, Catalog number 3957), determined using a 10-point standard curve (4PL fit) and CH58 mAb titration. Negative controls included NHS (HIV-1 seronegative human sera) and blank beads.

If the blank bead negative control exceeded 5000 MFI, the sample was repeated. If the repeat value exceeded 5000 MFI, the sample was excluded from the analysis due to high background. The net MFI, obtained by subtracting the blank bead responses, was used to summarize the magnitude at the specified dilutions. Net MFI values less than 1 were set equal to 1.

**Intracellular Cytokine Staining Assay (ICS)**

The average % of unstimulated, negative control cells was calculated by summing the number of CD4+ and CD8+ T cells expressing IFN-γ and/or IL-2 and the total count of CD4+ and CD8+ T cells over the two replicates and taking the ratio of these.

Background-adjusted percentages less than 0.01% were set equal to 0.01% (1/10,000). The following data were excluded from analysis: unreliable samples or visits outside allowable visit window. Records were excluded if the number of CD4+ T-cell subsets was <10,000 or the number of CD8+ T-cell subsets was <5,000. These criteria were applied separately to CD4+ and CD8+ subsets.

Positive responses: For determining positive responses, the four entries in each table are the number of cells positive for IFN-γ and/or IL-2 and the number of cells negative for IFN-γ and/or IL-2, for both the stimulated and the negative control cells. If both negative control replicates are included, then the total number of total cells and the total number of positive cells was used. Individual peptide-pool-specific p-values were adjusted using the discrete Bonferroni-Holm adjustment method to account for multiplicity over the number of peptide pools considered in this analysis. If the adjusted p-value was ≤ 0.00001, the response to the peptide pool for the corresponding T-cell subset was considered positive.

Magnitudes: For the non-overlapping Env ZM96-1 and Env ZM96-2 peptide pools, the magnitude for Env ZM96 was calculated as the sum of Env ZM96-1 and Env ZM96-2. Both pools were included regardless of positive or negative responder status and magnitudes less than zero were truncated at zero prior to summation. The overall magnitude was calculated as the sum of the individual Env ZM96-1 and Env ZM96-2 magnitudes, and overall ZM96 response was defined as positive if either Env ZM96-1 or Env ZM96-2 responses were positive. Negative magnitudes were censored at 0 prior to calculating the sum; if the sum is < 0.01%, the sum was set equal to 0.01%.

**Phylogenetic and Sequence Diversity Analyses**

***Methods:***

We used phylogenetic analyses to characterize the overall diversity of the vaccine inserts and antigens tested across all 36 trial regimens relative to HIV-1 Env global diversity ^1^ in past and circulating viruses. To this end, we compared a total of 12 vaccine inserts and 30 gp70 V1V2 antigen sequences (spanning Env HXB2 positions 120-213) to 4,676 env sequences downloaded from the LANL database and sampled from 1979 through 2021 (corresponding to all HIV-1 sequences that were deposited to GenBank in this time frame) and covering the major global circulating subtypes.

We extracted the gp70 V1V2 region (HXB2 positions 120-213), and merged and aligned this region with the 29 unique V1V2 antigen sequences and 11 vaccine inserts. One sequence, 92TH023, was both a vaccine insert and an antigen and was represented only once in the final alignment. Antigen 1086, and its N156Q/293F mutated version, were also represented only once in the tree as their sequences in the gp70 region are identical. For the final trees, hypervariable regions were excluded. Midpoint rooted, amino acid phylogenetic trees were created using the FastTree software^2,3^ with the JTT+CAT model and then visualized in R using the packages ape and phytools. Branch lengths were compared using Wilcoxon tests. V1V2 variable region characteristics were also computed using R and defined as follows: within HXB2 positions 130 and 196, we calculated V1V2 length to be the total number of amino acid residues; the number of glycosylation sites to be the total number of asparagine residues (N) followed by any residue except proline, followed by either a serine or threonine (i.e. the N[!P]S|T motif); and the total net charge was calculated by adding a positive charge for every K, R or H residues, and a negative charge for every D or E residue. Statistical comparisons were done by 2-sided Wilcoxon tests.

***Supplementary Results:***

Global midpoint-rooted phylogenetic tree (Figure S10): In contrast to subtype C and CRF01_AE antigens and vaccine inserts, subtype B antigens and vaccine inserts were mostly concentrated in the B subclade closer to the tree root. No antigen was found in the lower left quadrant of the subtype B clade, including two unique subgroups comprising B clade envs from Peru.

Sequence branch length (Figure S11): Older sequences had shorter branch lengths compared to circulating and more recently sampled sequences, consistent with expanding diversity over time. Despite being sampled between 1988 and 2007, we found that, on average, the 30 antigen sequences did not have shorter branch lengths compared to the full set of V1V2 sequences (Figure S11).

**Table S1. Heterologous V1V2 antigens assessed within each trial to evaluate IgG binding antibody responses.** All binding antibody multiplex assays run at 1:50 dilution. Red X’s denote antigens inducing the highest responses that are used in the IgG breadth score from each trial. As breadth scores are calculated by regimen, based on the 3 V1V2 antigens with the highest median responses among all participants, some trials may have more than 3 red X’s. Asterisks denote clade B, C or CRF07_BC antigens that were used in the IgG clade B+C breadth score.

| **Antigen** | **Clade** | **HVTN 096** | **HVTN 097** | **HVTN 100** | **HVTN 105** | **HVTN 106** | **HVTN 107** | **HVTN 108** | **HVTN 111** | **HVTN 120** | **HVTN 124** | **HVTN 702** | **HVTN 705** | **RV144** |
| --- | --- | --- | --- | --- | --- | --- | --- | --- | --- | --- | --- | --- | --- | --- |
| gp70_B.CaseA V1V2 | B | X* | X | X | X* | X* | X* | X* | X* | X* | X | X* | X* | X |
| gp70_B.CaseA2 V1/V2/169K | B | X* |  | X* | X* |  | X* |  |  | X* |  | X* |  | X |
| gp70-RHPA4259.7 V1V2 | B |  | X | X |  |  |  |  | X |  | X |  |  | X |
| gp70-TT31P.2F10.2792 V1V2 | B |  | X | X |  |  |  |  | X |  | X |  |  | X |
| gp70-1012.11.TC21.3257 V1V2 | B |  |  |  |  |  |  |  |  |  |  |  | X |  |
| gp70-1051.12.C22 V1V2 | B |  |  |  |  |  |  |  | X |  |  |  |  |  |
| gp70-62357.14 V1V2 | B |  | X | X |  |  |  |  | X |  | X |  |  | X |
| gp70-700010058 V1V2 | B |  | X | X |  |  |  |  | X |  | X* |  |  | X |
| gp70-96ZM651.02 V1v2 | C |  | X* |  |  |  |  |  |  |  | X* |  |  | X |
| C.1086C_V1_V2 Tags | C |  |  |  | X* | X* |  |  |  |  |  |  | X | X* |
| C.1086C_V1_V2_TagsN156Q/293F | C |  |  |  |  | X* |  |  |  |  |  |  |  |  |
| gp70_C.1086C V1/V2/293F | C |  |  |  |  |  |  |  |  |  | X* |  | X* | X* |
| gp70-TV1.21 V1V2 | C |  | X |  |  |  |  |  |  |  | X |  |  | X |
| gp70-001428.2.42 V1V2 | C |  | X | X |  |  |  |  | X |  | X |  | X | X |
| gp70-1394C9G1 V1V2 | C |  |  |  |  |  |  |  | X |  |  |  | X |  |
| gp70-7060101641 V1V2 | C |  | X* | X |  |  |  |  | X |  | X |  |  | X |
| gp70-BF1266_431a_V1V2 | C |  | X | X* |  |  |  |  | X* |  | X |  | X* | X |
| gp70-CAP210.2.00.E8 V1V2 | C |  | X | X |  |  |  |  |  |  | X |  |  | X |
| gp70-CAP45.2.00.G3 V1V2 | C |  |  |  |  |  |  |  | X |  |  |  |  |  |
| gp70-Ce1176 V1V2 | C |  |  |  |  |  |  |  | X |  |  |  |  |  |
| gp70-ConC V1V2 | C |  |  |  |  |  |  |  | X |  |  |  |  |  |
| gp70-Du156.12 V1V2 | C |  |  |  |  |  |  |  | X* |  |  |  |  |  |
| gp70-BJOX002000.03.2 V1V2 | CRF07_BC |  | X* | X* |  |  |  |  | X* |  | X* |  |  | X* |
| gp70-191084_B7 V1V2 | A1 |  | X | X |  |  |  |  | X |  | X |  |  | X |
| gp70-9004SS_A3_4 V1V2 | A1 |  |  |  |  |  |  |  |  |  |  |  | X |  |
| gp70-C2101.c01_V1V2 | CRF01_AE |  | X | X |  |  |  |  | X |  | X |  |  | X |
| AE.A244 V1V2 Tags/293F | CRF01_AE |  |  | X |  | X | X |  |  |  |  | X | X |  |
| AE.A244 V1V2_Tags_N156QN160Q /293F | CRF01_AE |  |  |  |  | X |  |  |  |  |  |  |  |  |
| gp70-CM244.ec1 V1V2 | CRF01_AE |  |  | X |  |  |  |  | X |  | X |  |  | X |
| gp70-92TH023 V1V2 | CRF01_AE |  |  |  |  |  |  |  |  |  |  | X |  |  |

**Table S2. Heterologous V1V2 antigens assessed within each trial to evaluate IgG3 binding antibody responses.** All binding antibody multiplex assays run at 1:40 dilution apart from HVTN 096, run at 1:50. Red X’s denote antigens used in the IgG3 breadth score from each trial. Asterisks denote clade B, C or CRF07_BC antigens that were used in the IgG3 clade B+C breadth score.

| **Antigen** | **Clade** | **HVTN 096** | **HVTN 097** | **HVTN 100** | **HVTN 105** | **HVTN 106** | **HVTN 107** | **HVTN 108** | **HVTN 111** | **HVTN 120** | **HVTN 124** | **HVTN 702** | **HVTN 705** | **RV144** |
| --- | --- | --- | --- | --- | --- | --- | --- | --- | --- | --- | --- | --- | --- | --- |
| gp70_B.CaseA V1V2 | B | X* | X* | X* | X* |  | X* | X* |  |  | X | X* | X | X |
| gp70_B.CaseA2 V1/V2/169K | B | X* |  | X* | X* |  | X* |  |  |  |  | X* |  | X* |
| gp70-1012.11.TC21.3257 V1V2 | B |  |  |  |  |  |  |  |  |  |  |  | X |  |
| gp70-62357.14 V1V2 | B |  |  |  |  |  |  |  |  |  | X |  |  |  |
| gp70-700010058 V1V2 | B |  |  |  |  |  |  |  |  |  | X |  |  |  |
| gp70-RHPA4259.7 V1V2 | B |  |  |  |  |  |  |  |  |  | X |  |  |  |
| gp70-TT31P.2F10.2792 V1V2 | B |  |  |  |  |  |  |  |  |  | X |  |  |  |
| gp70-96ZM651.02 V1v2 | C |  |  |  |  |  |  |  |  |  | X* |  |  | X* |
| C.1086C_V1_V2 Tags | C |  | X* |  | X* |  |  |  |  |  |  |  | X* | X* |
| gp70_C.1086C V1/V2/293F | C |  |  |  |  |  |  |  |  |  | X* |  | X* |  |
| gp70-TV1.21 V1V2 | C |  |  |  |  |  |  |  |  |  | X |  |  | X |
| gp70-001428.2.42 V1V2 | C |  |  |  |  |  |  |  |  |  | X* |  | X* |  |
| gp70-1394C9G1 V1V2 | C |  |  |  |  |  |  |  |  |  |  |  | X |  |
| gp70-7060101641 V1V2 | C |  |  |  |  |  |  |  |  |  | X |  |  |  |
| gp70-BF1266_431a_V1V2 | C |  |  |  |  |  |  |  |  |  | X |  | X |  |
| gp70-CAP210.2.00.E8 V1V2 | C |  |  |  |  |  |  |  |  |  | X |  |  |  |
| gp70-BJOX002000.03.2 V1V2 | CRF07_BC |  |  |  |  |  |  |  |  |  | X* |  |  |  |
| gp70-191084_B7 V1V2 | A1 |  |  |  |  |  |  |  |  |  | X |  |  |  |
| AE.A244 V1V2 Tags/293F | CRF01_AE |  |  | X |  |  | X |  |  |  |  | X | X |  |
| gp70-CM244.ec1 V1V2 | CRF01_AE |  |  |  |  |  |  |  |  |  | X |  |  |  |
| gp70-C2101.c01_V1V2 | CRF01_AE |  |  |  |  |  |  |  |  |  | X |  |  |  |

**Table S3. Information on the source isolate and virus of antigens reported in this manuscript.**

| **Antigen Name in this Manuscript** | **Source HIV-1 Isolate** | **Information on Source Virus** | **Note** |
| --- | --- | --- | --- |
| gp70-191084_B7 V1V2 | HIV-1 A1.191084.B7.19 | A1.UG.07.4 |  |
| gp70-TT31P.2F10.2792 V1V2 | HIV-1 B.TT31P.2F10 | B.TT.98.2 |  |
| gp70-RHPA4259.7 V1V2 | HIV-1 B.RHPA4259.7 | B.US.00.5 |  |
| gp70-700010058 V1V2 | HIV-1 B.7000010058.A4 | B.US.06.3 |  |
| gp70_B.CaseA V1V2 | HIV-1 B.CaseA.2 | B.US.88.6 |  |
| gp70_B.CaseA2 V1/V2/169K | HIV-1 B.CaseA.2/V169K | B.US.88.6 |  |
| gp70-62357.14 V1V2 | HIV-1 B.62357-14.D3 | B.US.96.2 | -14 refers to time point. |
| gp70-1051.12.C22 V1V2 | HIV-1 B.1051-12.C22 | B.US.97.2 | -12 refers to time point. |
| gp70-1012.11.TC21.3257 V1V2 | HIV-1 B.1012-11.TC21 | B.US.97.3 | -11 refers to time point. |
| gp70-001428.2.42 V1V2 | HIV-1 C.001428.2.42 | C.IN.00.4 |  |
| gp70-BF1266_431a_V1V2 | HIV-1 C.BF1266.431a | C.MW.02.1-2 |  |
| C.1086C_V1_V2 Tags | HIV-1 C.1086.B2 | C.MW.04.1-2 |  |
| gp70_C.1086C V1/V2/293F | HIV-1 C.1086.B2 | C.MW.04.1-2 |  |
| C.1086C_V1_V2_TagsN156Q/293F | HIV-1 C.1086.B2/N156Q | C.MW.04.1-2 |  |
| gp70-Ce1176 V1V2 | HIV-1 C.1176.A3 | C.MW.04.1-2 |  |
| gp70-1394C9G1 V1V2 | HIV-1 C.1394.C9.G1 | C.MW.04.1-2 |  |
| gp70-ConC V1V2 | HIV-1 C.Con | C.UNK.UNK.UNK | This is Con-C. |
| gp70-9004SS_A3_4 V1V2 | HIV-1 A1.9004SS.A3.4 | A1.UG.07.4 |  |
| gp70-CAP210.2.00.E8 V1V2 | HIV-1 C.CAP210.2.00.E8 | C.ZA.05.4 |  |
| gp70-CAP45.2.00.G3 V1V2 | HIV-1 C.Cap45.2.00.G3 | C.ZA.05.4 |  |
| gp70-7060101641 V1V2 | HIV-1 C.706010164.1A7 | C.ZA.07.4 |  |
| gp70-TV1.21 V1V2 | HIV-1 C.TV1.21 | C.ZA.98.6 | Not the vaccine isolate. |
| gp70-Du156.12 V1V2 | HIV-1 C.DU156.12 | C.ZA.99.1-4 | Note “DU” in all caps. |
| gp70-96ZM651.02 V1v2 | HIV-1 C.96ZM651.2 | C.ZM.96.6 |  |
| AE.A244 V1V2 Tags/293F | HIV-1 01AE.CM244.A244 | CRF01_AE.TH.90.6 | Note relationship between A244 and CM244. |
| AE.A244 V1V2_Tags_N156QN160Q /293F | HIV-1 01AE.CM244.A244/N156Q.N160Q | CRF01_AE.TH.90.6 |  |
| gp70-CM244.ec1 V1V2 | HIV-1 01AE.CM244.ec1 | CRF01_AE.TH.90.6 |  |
| gp70-92TH023 V1V2 | HIV-1 01AE.TH023.6/K109E or HIV-01AE.TH023.vCP1521 | CRF01_AE.TH.92.6 | This antigen has a mutation relative to TH023.6 (K109E) - the mutation makes it identical to the vaccine strain vCP1521 |
| gp70-C2101.c01_V1V2 | HIV-1 01AE.C2101.c01 | CRF01_AE.TH.99.UNK |  |
| gp70-BJOX002000.03.2 V1V2 | HIV-1 07BC.BJOX002000.03.2 | CRF07_BC.CN.07.1-2 | Can use BJOX002.03.2. |
| Con 6 gp120/B | HIV-1 M.Con-6 | Group M.UNK.UNK.UNK |  |

**Table S4. HIV peptide pools assessed within each trial to evaluate CD4+ and CD8+ T cell responses by intracellular cytokine staining.** Vaccine-matched peptide pools are indicated by *. ENV-1-PTEG, ENV-PTEG-SEQ, ENV-2-PTEG, ENV-2-PTEG-SEQ, and ENV-3-PTEG are different global potential T-cell HIV-1 Env epitope peptide pools representing 15-mer peptides.^4^ Gag-1-PTEG-SEQ and Gag-2-PTEG-SEQ are different global potential T-cell HIV-1 Gag epitope peptide pools.

| **Protocol** | **Any Env** | | | | | **Any Gag** | |
| --- | --- | --- | --- | --- | --- | --- | --- |
| HVTN 096 | Env-1-ZM96* | Env-2-ZM96* |  |  |  | Gag-ZM96* |  |
| HVTN 097 | ZM96 gp120* | 1086 gp120* | TV1 gp120* | 92TH023-ENV* |  |  |  |
| HVTN 100 | ZM96 gp120* | 1086 gp120* | TV1 gp120* |  |  | LAI Gag* |  |
| HVTN 105 | Env-1-ZM96* | Env-2-ZM96* | 92TH023-ENV* |  |  | Gag-ZM96* |  |
| HVTN 106 | ConS gp120 | ConS gp41 | ENV-1-PTEG | ENV-2-PTEG | ENV-3-PTEG |  |  |
| HVTN 107 | Env-1-ZM96* | Env-2-ZM96* | 1086 gp120* | TV1 gp120* |  | LAI Gag* |  |
| HVTN 108 | Env-1-ZM96* | Env-2-ZM96* | 1086 gp120* | TV1 gp120* |  | Gag-ZM96* |  |
| HVTN 111 | Env-1-ZM96* | Env-2-ZM96* | 1086 gp120* | TV1 gp120* |  | Gag-ZM96* |  |
| HVTN 120 | Env-1-ZM96* | Env-2-ZM96* | 1086 gp120* | TV1 gp120* |  | LAI Gag* |  |
| HVTN 124 | Env-1-PTEG-SEQ | Env-2-PTEG-SEQ |  |  |  | Gag-1-PTEG-SEQ | Gag-2-PTEG-SEQ |
| HVTN 702 | ZM96 gp120* | 1086 gp120* | TV1 gp120* |  |  | LAI Gag* |  |
| HVTN 705 | J Mos1 gp120* | J Mos1 gp 41* | J Mos2S gp120* | J Mos2S gp41* |  | J Mos2 Gag* |  |
| RV144 | 92TH023-ENV* |  |  |  |  |  |  |

*Vaccine-matched peptide pools.

**Table S5.** **Baseline demographics for participants with immunogenicity data.**

| **Characteristics** | **HVTN 096** | **HVTN 097** | **HVTN 100** | **HVTN 105** | **HVTN 106** | **HVTN 107** | **HVTN 108** | **HVTN 111** | **HVTN 120** | **HVTN 124** | **HVTN 702** | **HVTN 705** | **RV144** | **Total** |
| --- | --- | --- | --- | --- | --- | --- | --- | --- | --- | --- | --- | --- | --- | --- |
| N | 67 | 73 | 186 | 102 | 73 | 107 | 264 | 115 | 140 | 30 | 60 | 270 | 201 | 1688 |
| Sex |  |  |  |  |  |  |  |  |  |  |  |  |  |  |
| Female | 30 (44.8%) | 33 (45.2%) | 74 (39.8%) | 48 (47.1%) | 31 (42.5%) | 60 (56.1%) | 128 (48.5%) | 60 (52.2%) | 78 (55.7%) | 20 (66.7%) | 60 (100.0%)^1^ | 270 (100.0%)^2^ | 79 (39.3%) | 971 (57.5%) |
| Male | 37 (55.2%) | 40 (54.8%) | 112 (60.2%) | 54 (52.9%) | 42 (57.5%) | 47 (43.9%) | 136 (51.5%) | 55 (47.8%) | 62 (44.3%) | 10 (33.3%) |  |  | 122 (60.7%) | 717 (42.5%) |
| Race |  |  |  |  |  |  |  |  |  |  |  |  |  |  |
| Asian | 1 (1.5%) | 0 (0%) | 1 (0.5%) | 5 (4.9%) | 5 (6.8%) | 0 (0%) | 16 (6.1%) | 0 (0%) | 2 (1.4%) | 3 (10.0%) | 0 (0%) | 0 (0%) | NA^3^ | 33 (2.2%) |
| Black/African American | 2 (3.0%) | 73 (100.0%) | 182 (97.8%) | 14 (13.7%) | 15 (20.5%) | 107 (100.0%) | 130 (49.2%) | 114 (99.1%) | 88 (62.9%) | 3 (10.0%) | 59 (98.3%) | 266 (98.5%) | NA^3^ | 1053 (70.8%) |
| Native American/ Alaskan Native | 0 (0%) | 0 (0%) | 0 (0%) | 1 (1.0%) | 0 (0%) | 0 (0%) | 1 (0.4%) | 0 (0%) | 0 (0%) | 0 (0%) | 0 (0%) | 0 (0%) | NA^3^ | 2 (0.1%) |
| White | 61 (91.0%) | 0 (0%) | 0 (0%) | 71 (69.6%) | 47 (64.4%) | 0 (0%) | 99 (37.5%) | 0 (0%) | 44 (31.4%) | 21 (70.0%) | 0 (0%) | 1 (0.4%) | NA^3^ | 344 (23.1%) |
| Multiracial | 0 (0%) | 0 (0%) | 0 (0%) | 9 (8.8%) | 5 (6.8%) | 0 (0%) | 9 (3.4%) | 0 (0%) | 6 (4.3%) | 2 (6.7%) | 1 (1.7%) | 3 (1.1%) | NA^3^ | 35 (2.4%) |
| Other | 3 (4.5%) | 0 (0%) | 3 (1.6%) | 2 (2.0%) | 1 (1.4%) | 0 (0%) | 9 (3.4%) | 1 (0.9%) | 0 (0%) | 1 (3.3%) | 0 (0%) | 0 (0%) | NA^3^ | 20 (1.3%) |
| Ethnicity |  |  |  |  |  |  |  |  |  |  |  |  |  |  |
| Hispanic or Latino | 14 (20.9%) | 0 (0%) | 0 (0%) | 10 (9.8%) | 4 (5.5%) | 0 (0%) | 21 (8.0%) | 0 (0%) | 3 (2.1%) | 1 (3.3%) | NA | 0 (0%) | NA^3^ | 53 (3.7%) |
| Not Hispanic or Latino | 53 (79.1%) | 73 (100.0%) | 186 (100.0%) | 92 (90.2%) | 69 (94.5%) | 107 (100.0%) | 243 (92.0%) | 115 (100.0%) | 137 (97.9%) | 29 (96.7%) | NA | 270  (100.0%) | NA^3^ | 1374 (96.3%) |
| Age |  |  |  |  |  |  |  |  |  |  |  |  |  |  |
| 18-20 | 5 (7.5%) | 27 (37.0%) | 44 (23.7%) | 11 (10.8%) | 8 (11.0%) | 14 (13.1%) | 42 (15.9%) | 28 (24.3%) | 20 (14.3%) | 3 (10.0%) | 10 (16.7%) | 79 (29.3%) | 56 (27.9%) | 347 (20.6%) |
| 21-25 | 25 (37.3%) | 29 (39.7%) | 78 (41.9%) | 31 (30.4%) | 13 (17.8%) | 40 (37.4%) | 104 (39.4%) | 45 (39.1%) | 64 (45.7%) | 12 (40.0%) | 35 (58.3%) | 128 (47.4%) | 97 (48.3%) | 701 (41.5%) |
| 26-30 | 13 (19.4%) | 14 (19.2%) | 44 (23.7%) | 25 (24.5%) | 15 (20.5%) | 39 (36.4%) | 76 (28.8%) | 30 (26.1%) | 27 (19.3%) | 2 (6.7%) | 11 (18.3%) | 45 (16.7%) | 48 (23.9%) | 389 (23.0%) |
| 31-35 | 13 (19.4%) | 3 (4.1%) | 16 (8.6%) | 13 (12.7%) | 8 (11.0%) | 7 (6.5%) | 26 (9.8%) | 4 (3.5%) | 19 (13.6%) | 9 (30.0%) | 4 (6.7%) | 18 (6.7%) | 0 (0%) | 140 (8.3%) |
| 36-40 | 5 (7.5%) | 0 (0%) | 4 (2.2%) | 7 (6.9%) | 7 (9.6%) | 7 (6.5%) | 16 (6.1%) | 8 (7.0%) | 10 (7.1%) | 3 (10.0%) | 0 (0%) | 0 (0%) | 0 (0%) | 67 (4.0%) |
| 41-50 | 6 (9.0%) | 0 (0%) | 0 (0%) | 15 (14.7%) | 22 (30.1%) | 0 (0%) | 0 (0%) | 0 (0%) | 0 (0%) | 1 (3.3%) | 0 (0%) | 0 (0%) | 0 (0%) | 44 (2.6%) |
| BMI |  |  |  |  |  |  |  |  |  |  |  |  |  |  |
| Min | 18.6 | 16.8 | 15.8 | 19 | 19.1 | 17.6 | 15.1 | 17.3 | 16.2 | 20.1 | 16.6 | 15.7 | NA | 15.1 |
| 25% | 21.7 | 19.2 | 20 | 22.4 | 22.6 | 20.8 | 21.5 | 20.6 | 20.6 | 23.5 | 21.8 | 20.7 | NA | 21 |
| Median | 23.5 | 21.1 | 22.4 | 26 | 25 | 23.6 | 23.9 | 22.6 | 22.8 | 25.4 | 25.4 | 23.3 | NA | 23.5 |
| 75% | 25.6 | 26.1 | 26.6 | 29.3 | 28.4 | 28.5 | 27.3 | 25.3 | 25.5 | 30.8 | 31.2 | 27.7 | NA | 27.4 |
| Max | 32 | 37.3 | 38.9 | 39.3 | 37.8 | 38.1 | 39.1 | 39.5 | 38.1 | 36 | 46.6 | 59.5 | NA | 59.5 |
| Country |  |  |  |  |  |  |  |  |  |  |  |  |  |  |
| Canada | 0 (0%) | 0 (0%) | 0 (0%) | 0 (0%) | 0 (0%) | 0 (0%) | 14 (5.3%) | 0 (0%) | 0 (0%) | 0 (0%) | 0 (0%) | 27 (10.0%) | 0 (0%) | 41 (2.4%) |
| Mozambique | 0 (0%) | 0 (0%) | 0 (0%) | 0 (0%) | 0 (0%) | 14 (13.1%) | 0 (0%) | 0 (0%) | 0 (0%) | 0 (0%) | 0 (0%) | 2 (0.7%) | 0 (0%) | 16 (0.9%) |
| South Africa | 0 (0%) | 73 (100.0%) | 186 (100.0%) | 0 (0%) | 0 (0%) | 65 (60.7%) | 97 (36.7%) | 65 (56.5%) | 0 (0%) | 0 (0%) | 60 (100.0%) | 178 (65.9%) | 0 (0%) | 724 (42.9%) |
| Switzerland | 67 (100.0%) | 0 (0%) | 0 (0%) | 0 (0%) | 12 (16.4%) | 0 (0%) | 0 (0%) | 0 (0%) | 0 (0%) | 0 (0%) | 0 (0%) | 0 (0%) | 0 (0%) | 79 (4.7%) |
| Tanzania | 0 (0%) | 0 (0%) | 0 (0%) | 0 (0%) | 0 (0%) | 0 (0%) | 0 (0%) | 25 (21.7%) | 42 (30.0%) | 0 (0%) | 0 (0%) | 0 (0%) | 0 (0%) | 67 (4.0%) |
| Thailand | 0 (0%) | 0 (0%) | 0 (0%) | 0 (0%) | 0 (0%) | 0 (0%) | 0 (0%) | 0 (0%) | 0 (0%) | 0 (0%) | 0 (0%) | 0 (0%) | 201 (100%) | 201 (11.9%) |
| United States | 0 (0%) | 0 (0%) | 0 (0%) | 102 (100.0%) | 61 (83.6%) | 0 (0%) | 153 (58.0%) | 0 (0%) | 54 (38.6%) | 30 (100.0%) | 0 (0%) | 0 (0%) | 0 (0%) | 400 (23.7%) |
| Zambia | 0 (0%) | 0 (0%) | 0 (0%) | 0 (0%) | 0 (0%) | 0 (0%) | 0 (0%) | 25 (21.7%) | 15 (10.7%) | 0 (0%) | 0 (0%) | 24 (8.9%) | 0 (0%) | 64 (3.8%) |
| Zimbabwe | 0 (0%) | 0 (0%) | 0 (0%) | 0 (0%) | 0 (0%) | 28 (26.2%) | 0 (0%) | 0 (0%) | 29 (20.7%) | 0 (0%) | 0 (0%) | 28 (10.4%) | 0 (0%) | 85 (5.0%) |
| Malawi | 0 (0%) | 0 (0%) | 0 (0%) | 0 (0%) | 0 (0%) | 0 (0%) | 0 (0%) | 0 (0%) | 0 (0%) | 0 (0%) | 0 (0%) | 11 (4.1%) | 0 (0%) | 11 (0.7%) |

NA = Not available.

^1^In HVTN 702, immune responses were only measured from females.

^2^In HVTN 705, only females were enrolled.

^3^In RV144, Thai men and women were recruited, but data on race/ethnicity was not collected. Hence, participants from RV144 were not included in the overall denominator when calculating % of race and ethnicity.

**Figure S1. Locations at which the 13 trials were conducted.** See also Table 1.

**
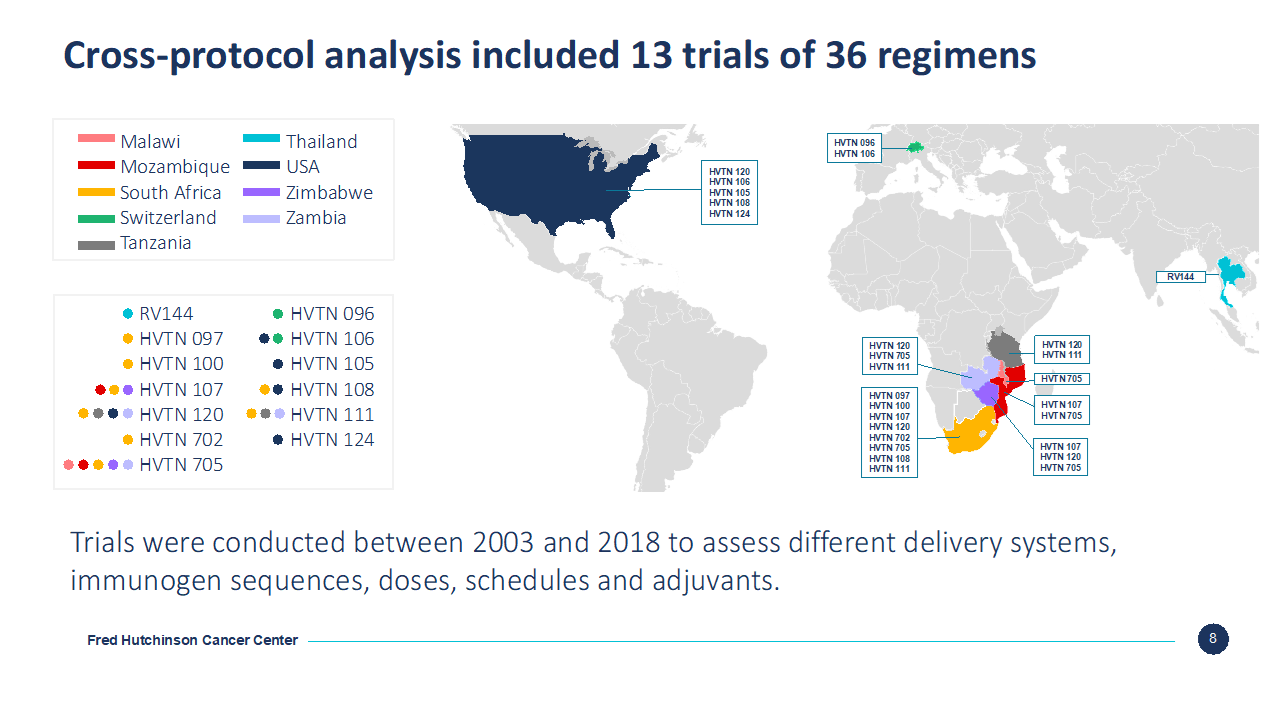
**

**Figure S2. (A, B) Distributions of binding antibody V1V2 heterologous clade B+C breadth scores in each regimen and (C, D) pairwise comparisons between regimens of binding antibody V1V2 heterologous clade B+C breadth score distributions.** The box plots show the distribution of (A) IgG and (B) IgG3 binding antibody V1V2 heterologous clade B+C breadth scores across participants in a given regimen, where each dot represents one participant. The horizontal line in each box represents the median regimen-specific breadth score. The number of participants (n) in each regimen is provided in the top row. Within a given regimen, the V1V2 heterologous clade B+C breadth score was defined as the geometric mean of the binding antibody responses to the heterologous clade B+C V1V2 antigens with the highest median responses among all participants in that regimen, denoted in Tables S1 and S2. The tile plots display the difference in medians for each regimen pair in the (C) IgG and (D) IgG3 binding antibody V1V2 heterologous clade B+C breadth score with asterisks denoting statistical significance: ** Wilcoxon test p-value < 0.001; * Wilcoxon test p-value < 0.05.

**
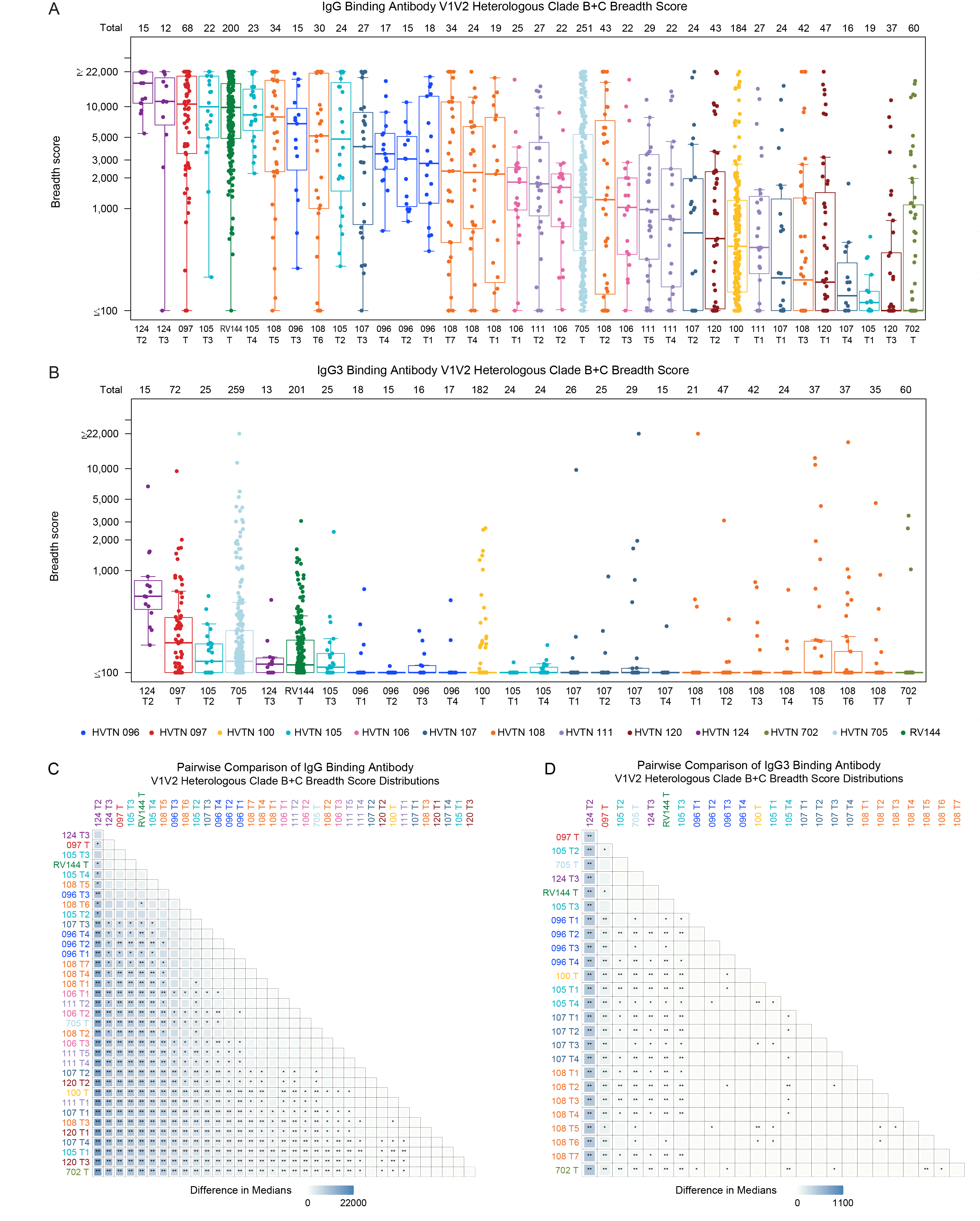
**

**Figure S3. CD4+ T-cell IFN-γ and/or IL-2 expression in response to Any Gag by trial: (A) response rates, (B) response magnitudes, (C) pairwise comparison between regimens of response rates, (D) pairwise comparison between regimens of response magnitude distributions.** The box plot in (B) shows the distribution of % CD4+ T cells expressing IFN-γ and/or IL-2 in response to Any Gag across participants in a given regimen, where each dot represents one participant. The horizontal line in each box represents the median regimen-specific % CD4+ T cells expressing IFN-γ and/or IL-2 in response to Any Gag. The number of participants (n) in each regimen is provided in the top row (Total). The tile plots in (C) and (D) display the difference for each regimen pair in (C) response rate and (D) median response magnitude, with asterisks denoting statistical significance. In (C): ** indicates Barnard’s test p-value < 0.001, * indicates p-value < 0.05. In (D): ** indicates Wilcoxon test p-value < 0.001, * indicates p-value < 0.05.


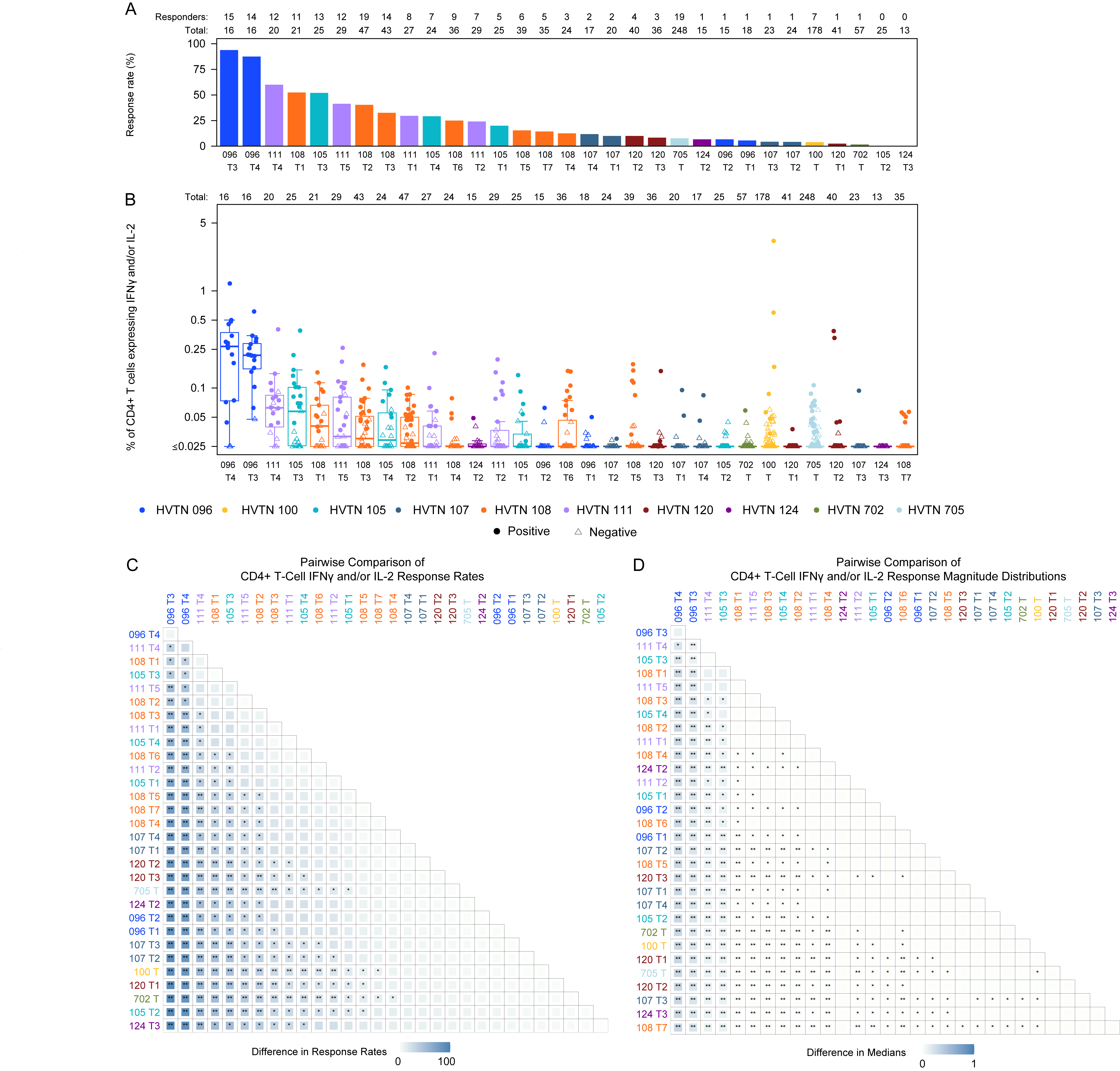


­

**Figure S4. IgG3 binding antibody responses to gp70_B.CaseA V1V2 by trial and comparisons between arms across trials. Dilution was 1:40 for all trials except for HVTN 096, which was run at 1:50.** IgG3 binding antibody responses to gp70_B.CaseA V1V2 by trial and comparisons between arms across trials: (A) response rates, (B) response magnitude distributions, (C) pairwise comparison between regimens of response rates, (D) pairwise comparison between regimens of response magnitude distributions. The box plot in (B) shows the distribution of IgG3 binding antibody response magnitudes to gp70_B.CaseA V1V2 across participants in a given regimen, where each dot represents one participant. The horizontal line in each box represents the median regimen-specific IgG3 gp70_B.CaseA V1V2 response magnitude. The number of participants (n) in each regimen is provided in the top row (Total). The tile plots in (C) and (D) display the difference for each regimen pair in (C) response rate and (D) median response magnitude, with asterisks denoting statistical significance. In (C): ** indicates Barnard’s test p-value < 0.001, * indicates p-value < 0.05 (D) magnitude comparison between arms across trials. In (D): ** indicates Wilcoxon test p-value < 0.001, * indicates p-value < 0.05.

**
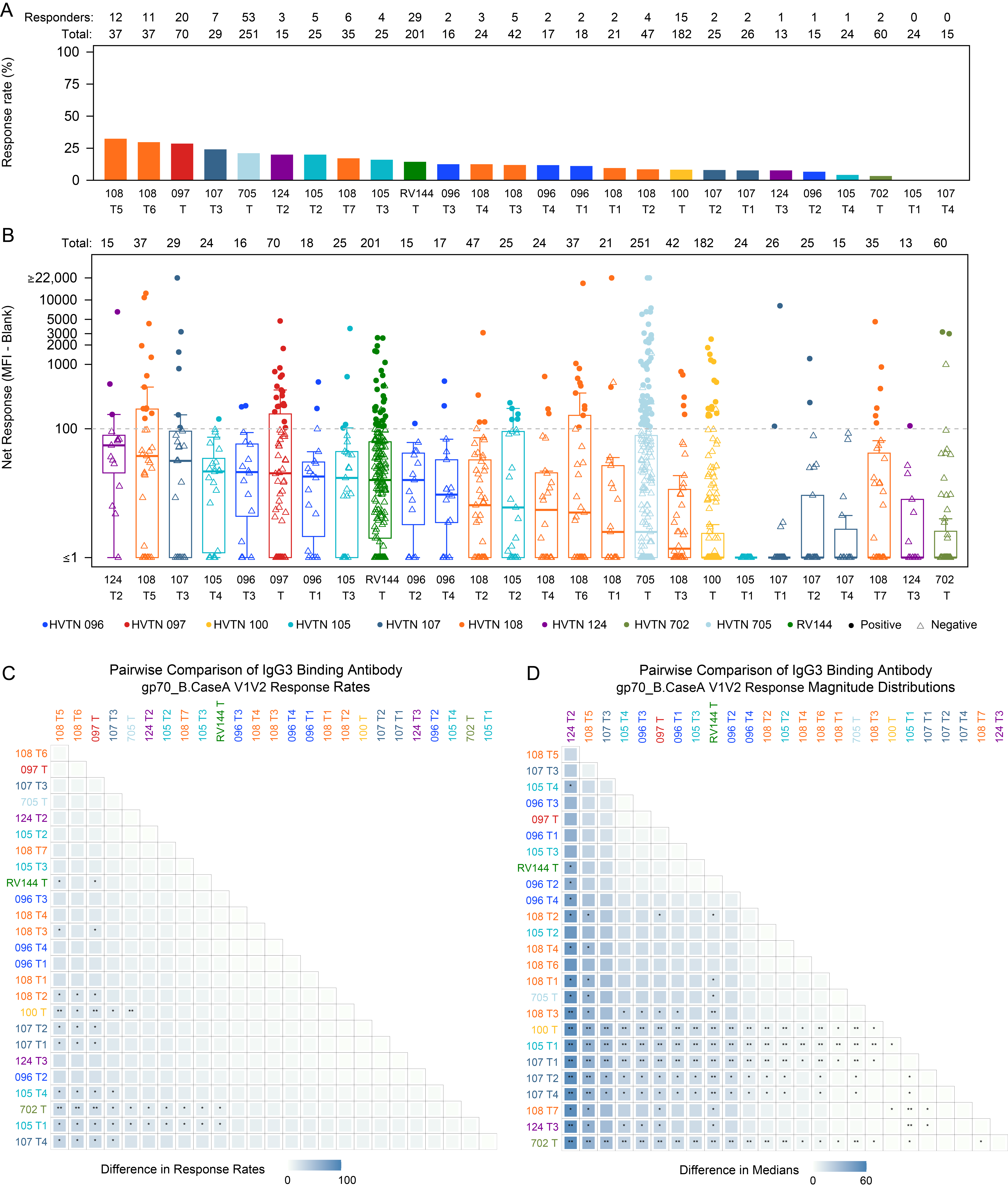
**

**Figure S5. CD8+ T-cell IFN-γ and/or IL-2 expression in response to Any Env by trial (A) response rates, (B) response magnitudes.** The box plot in (B) shows the distribution of % CD8+ T cells expressing IFN-γ and/or IL-2 in response to Any Env across participants in a given regimen, where each dot represents one participant. The horizontal line in each box represents the median regimen-specific % CD8+ T cells expressing IFN-γ and/or IL-2 in response to Any Env. The number of participants (n) in each regimen is provided in the top row (Total).

**
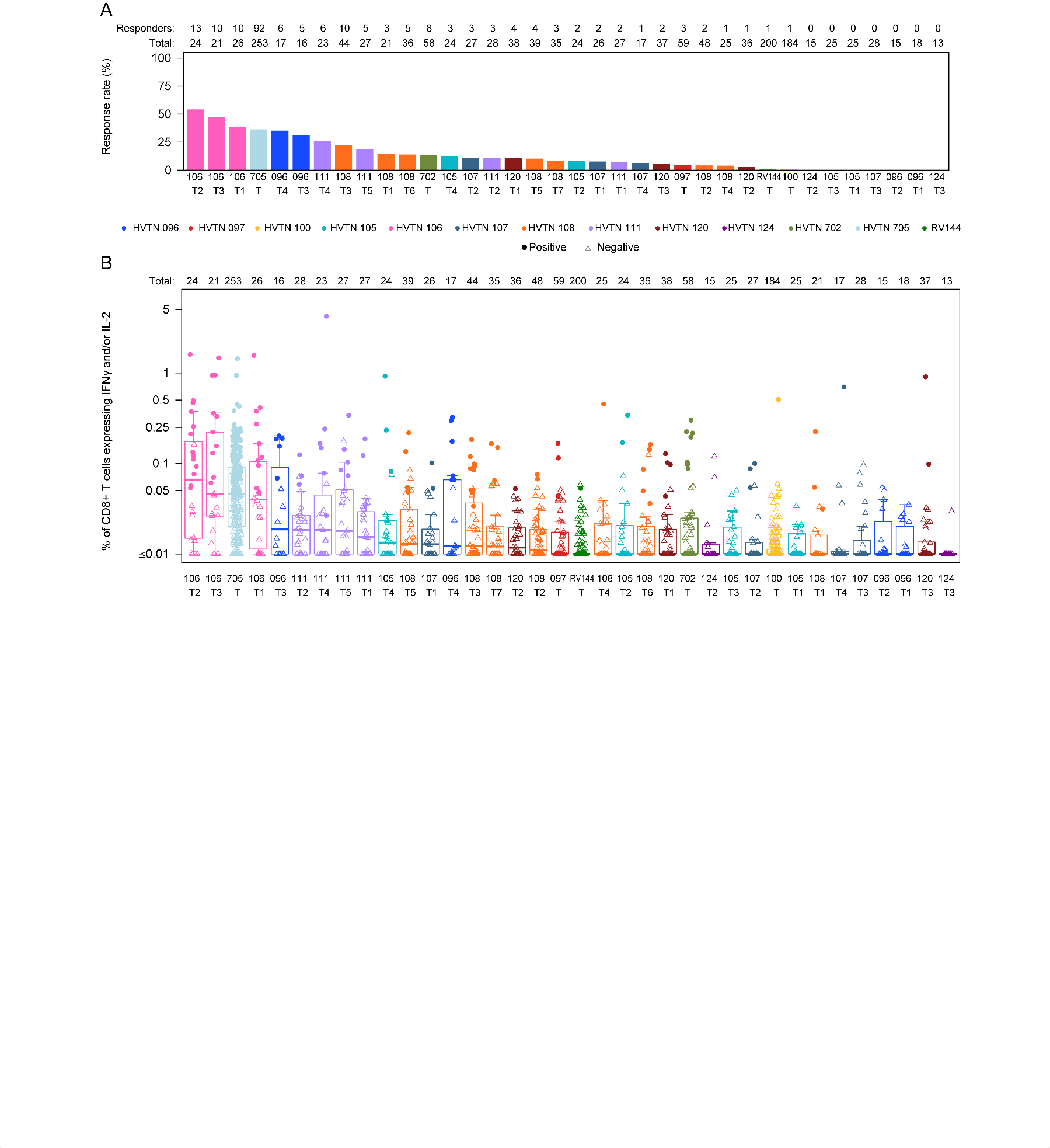
**

**Figure S6. CD8+ T-cell IFN-γ and/or IL-2 expression in response to Any Gag by trial (A) response rates, (B) response magnitudes.** The box plot in (B) shows the distribution of % CD8+ T cells expressing IFN-γ and/or IL-2 in response to Any Gag across participants in a given regimen, where each dot represents one participant. The horizontal line in each box represents the median regimen-specific % CD8+ T cells expressing IFN-γ and/or IL-2 in response to Any Gag. The number of participants (n) in each regimen is provided in the top row (Total).

**
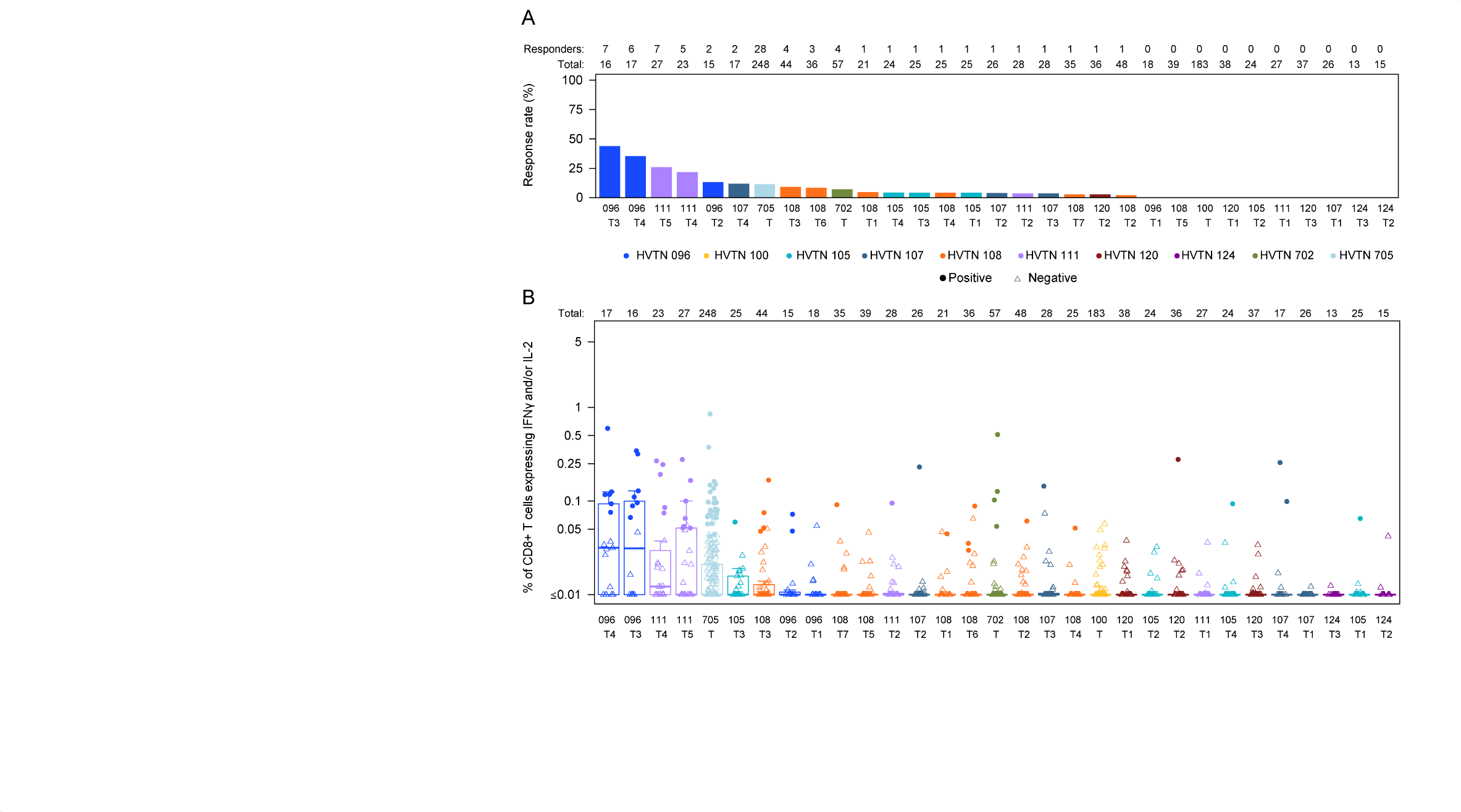
**

**Figure S7. IgG binding antibody responses to Con 6 gp120/B by trial and comparisons between arms across trials.** (A) response rates, (B) response magnitude distributions, (C) pairwise comparison between regimens of response rates, (D) pairwise comparison between regimens of response magnitude distributions. The box plot in (B) shows the distribution of IgG binding antibody response magnitudes to Con 6 gp120/B across participants in a given regimen, where each dot represents one participant. The horizontal line in each box represents the median regimen-specific IgG Con 6 gp120/B response magnitude. The number of participants (n) in each regimen is provided in the top row (Total). The tile plots in (C) and (D) display the difference for each regimen pair in (C) response rate and (D) median response magnitude, with asterisks denoting statistical significance. In (C): ** indicates Barnard’s test p-value < 0.001, * indicates p-value < 0.05 (D) magnitude comparison between arms across trials. In (D): ** indicates Wilcoxon test p-value < 0.001, * indicates p-value < 0.05. In (D), the absence of a square indicates that the comparison was not done due to a saturated median response (≥22,000 MFI) in at least one of the regimens.


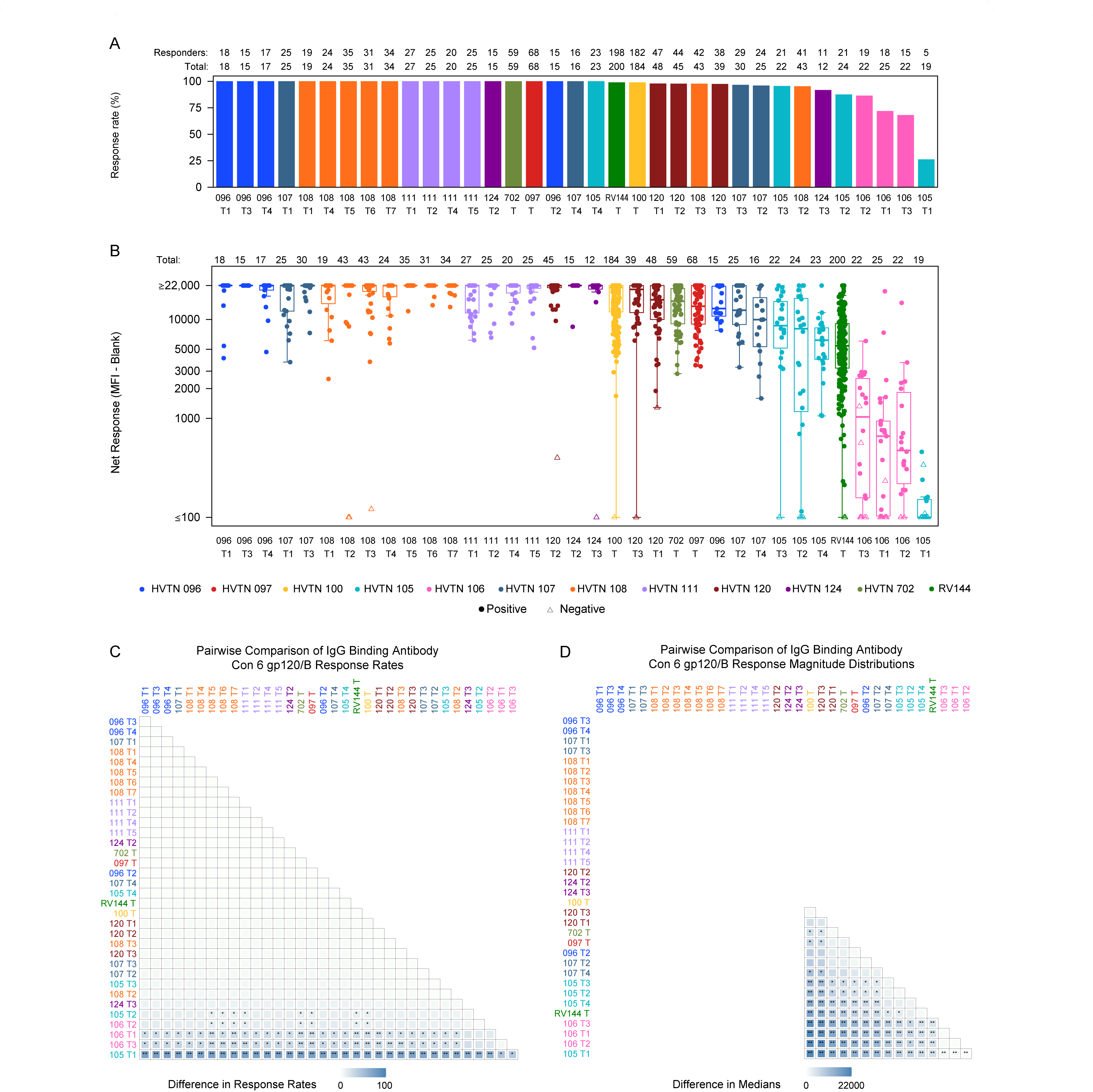


**Figure S8. IgG3 binding antibody responses to Con 6 gp120/B by trial and comparisons between arms across trials.** (A) response rates, (B) response magnitude distributions, (C) pairwise comparison between regimens of response rates, (D) pairwise comparison between regimens of response magnitude distributions. The box plot in (B) shows the distribution of IgG3 binding antibody response magnitudes to Con 6 gp120/B across participants in a given regimen, where each dot represents one participant. The horizontal line in each box represents the median regimen-specific IgG Con 6 gp120/B response magnitude. The number of participants (n) in each regimen is provided in the top row (Total). The tile plots in (C) and (D) display the difference for each regimen pair in (C) response rate and (D) median response magnitude, with asterisks denoting statistical significance. In (C): ** indicates Barnard’s test p-value < 0.001, * indicates p-value < 0.05 (D) magnitude comparison between arms across trials. In (D): ** indicates Wilcoxon test p-value < 0.001, * indicates p-value < 0.05.


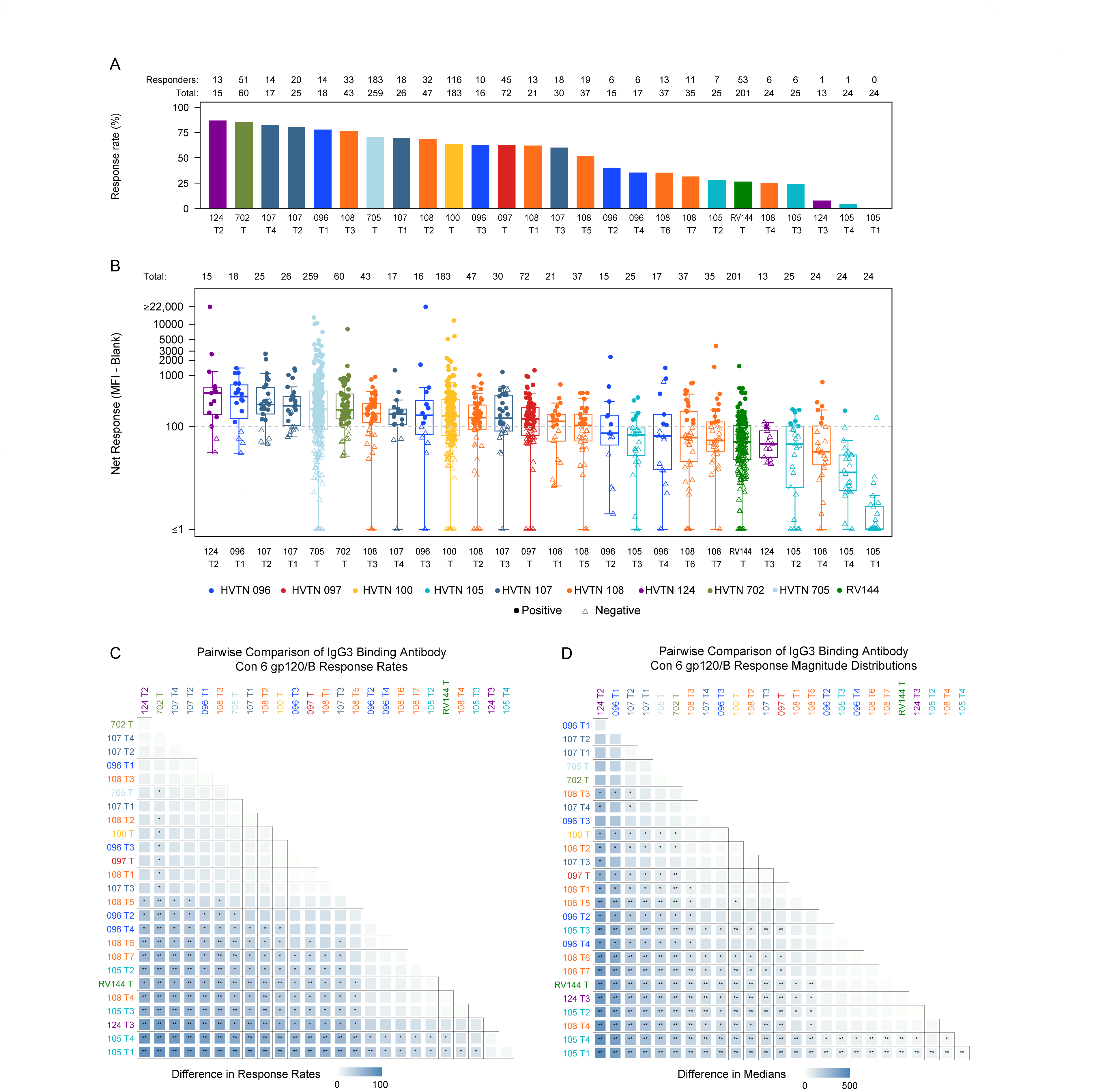


**Figure S9**. **Midpoint rooted phylogenetic tree of 29 unique V1V2 antigen sequences^5^ and 12 vaccine inserts.*** Vaccine inserts are shown in red, in different shapes, with vaccine inserts in the HVTN 702 regimen highlighted. Antigen V1V2 sequences are shown with filled circles, color-coded by clade. The tree was constructed from the amino acid alignment using PhyML, with the HIVb model for protein evolution and Gamma distribution. *The 92TH023 sequence is both a vaccine insert and an antigen and is labeled as both in the tree. Antigens 1086 and 1086.293F have identical gp70 sequences, hence they are represented once in the phylogenetic tree.





**Figure S10**. **Midpoint rooted phylogenetic tree of 30 V1V2 antigen sequences,^5^ 12 vaccine inserts,* and 4,676 V1V2 sequences from the LANL database sampled from 1979 through 2021.** Vaccine inserts are shown as red triangles, the V1V2 antigens as filled circles in magenta, and all database sequences are shown as open diamonds and color-coded by clade. The tree was constructed with FastTree from the amino acid alignment using the JTT+CAT model. Hypervariable regions were removed. *The 92TH023 sequence is both a vaccine insert and an antigen and is labeled as both in the tree. Antigens 1086 and 1086.293F have identical gp70 sequences, hence they are represented once in the phylogenetic tree.


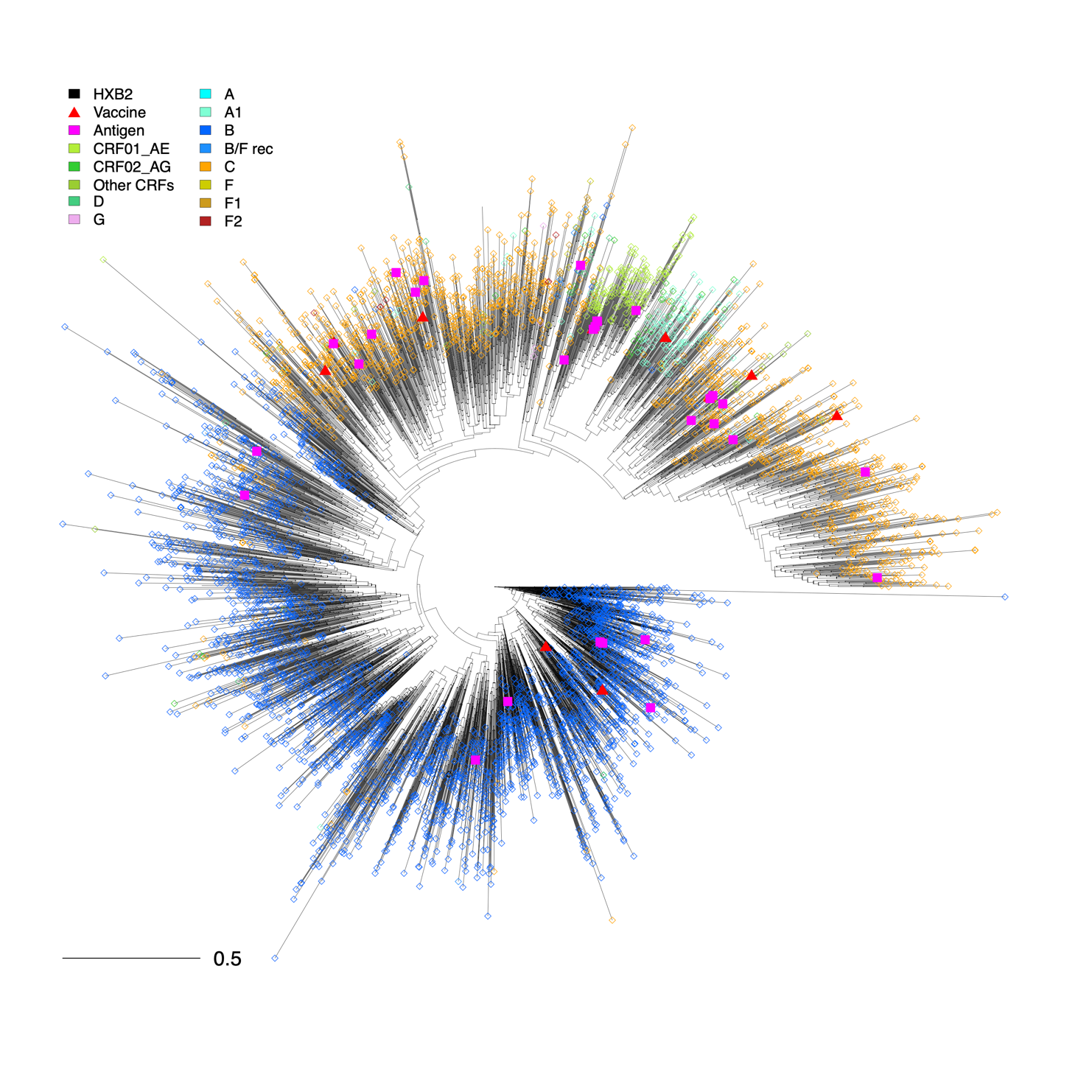


**Figure S11. V1V2 branch lengths by sampling year.** Branch lengths from the global midpoint rooted tree in Figure S10 are plotted by sampling year for the gp70 sequences in the tree for which sampling year data was available (4,338 of the 4,676 analyzed). Database sequence branch lengths are shown in light blue and branch lengths from the 30 antigen sequences are shown in magenta. Increasing linear fit is shown in the red dashed line. Correlation Tau shows a strongly significant trend (τ=0.08, p<0.0001).

**
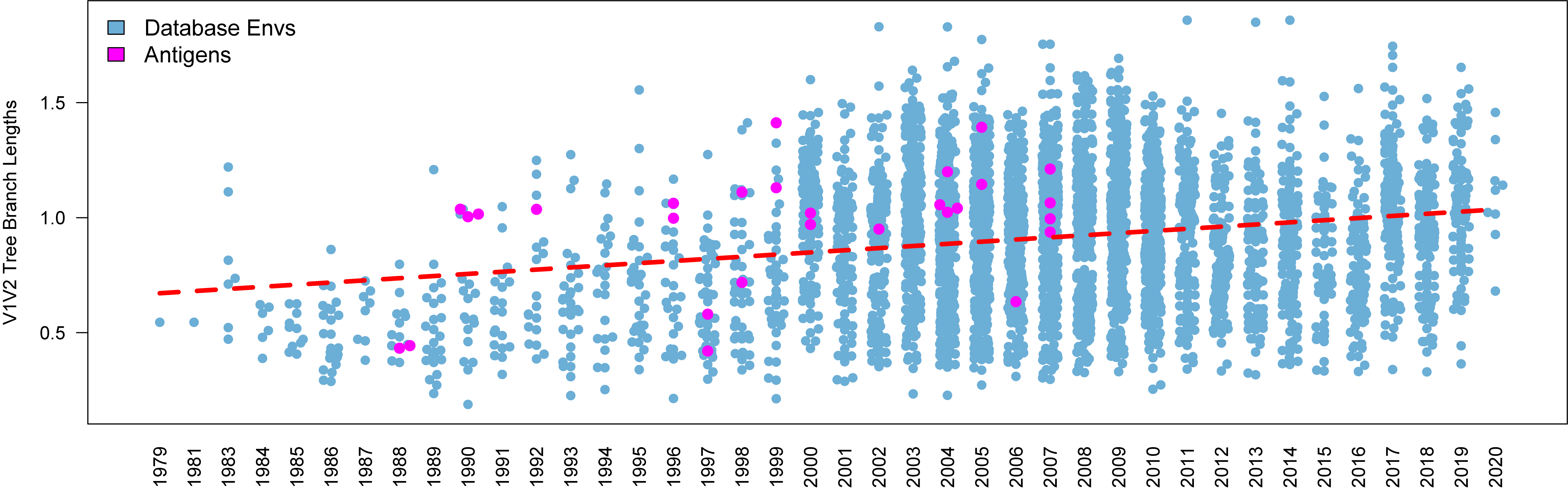
**

**Figure S12. V1V2 variable characteristics of 4,676 database envs compared to 30 antigen sequences.** Histograms in light blue show the distributions of the database env V1V2 characteristics, whereas the antigen characteristics distributions are shown in magenta. Black bars show the corresponding medians. The only significant comparison was for net charge: database envs had a statistically significantly lower median charge of –1.5 compared to the antigens’ median net charge of 0 (p=0.0016 by Wilcoxon test).


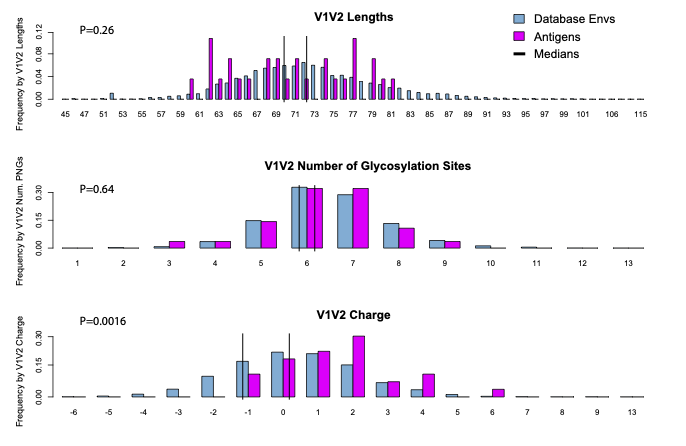


**Supplementary References**

1. Bbosa N, Kaleebu P, Ssemwanga D. HIV subtype diversity worldwide. *Curr Opin HIV AIDS* 2019; **14**(3): 153-60.

2. Price MN, Dehal PS, Arkin AP. FastTree: computing large minimum evolution trees with profiles instead of a distance matrix. *Mol Biol Evol* 2009; **26**(7): 1641-50.

3. Price MN, Dehal PS, Arkin AP. FastTree 2--approximately maximum-likelihood trees for large alignments. *PLoS One* 2010; **5**(3): e9490.

4. Li F, Malhotra U, Gilbert PB, et al. Peptide selection for human immunodeficiency virus type 1 CTL-based vaccine evaluation. *Vaccine* 2006; **24**(47-48): 6893-904.

5. Yates NL, deCamp AC, Korber BT, et al. HIV-1 Envelope Glycoproteins from Diverse Clades Differentiate Antibody Responses and Durability among Vaccinees. *J Virol* 2018; **92**(8).
